# Does population density moderate suicide risk? An Italian population study over the last 30 years

**DOI:** 10.1192/j.eurpsy.2020.69

**Published:** 2020-07-01

**Authors:** Monica Vichi, Benedetto Vitiello, Silvia Ghirini, Maurizio Pompili

**Affiliations:** 1 Statistical Service, Istituto Superiore di Sanità, Rome, Italy; 2 Department of Public Health and Pediatric Sciences, Università degli Studi di Torino, Turin, Italy; 3 Department of Mental Health, School of Public Health, Johns Hopkins University, Baltimore, Maryland, USA; 4 National Centre on Addiction and Substance Abuse, Istituto Superiore di Sanità, Rome, Italy; 5 Department of Neurosciences, Mental Health and Sensory Organs, Suicide Prevention Center, Sant’Andrea Hospital, Sapienza University of Rome, Rome, Italy

**Keywords:** Mortality, population density, sex, suicide

## Abstract

**Background.:**

The relationship between population density and suicide risk remains unclear. While urbanization is associated with greater risk for psychopathology, higher suicide rates have been reported in rural areas. We examined population density and suicide in the Italian population in the last 30 years.

**Methods.:**

The Italian National Institute of Statistics databases of the Italian population aged 15 years and older (52.4 million in 2016) were used to compute age-adjusted annual total mortality and suicide rates for the years 1985–2016. According to the European Union statistical office (EUROSTAT) criteria, municipalities were classified into *densely populated areas*, *intermediate density areas*, *or thinly populated areas*. Rate ratios (RRs) were computed by sex, age, and geographical area, using *densely populated areas* as reference.

**Results.:**

Total mortality was not associated with population density. In males, suicide rate increased with decreasing population density (RR = 1.17, 95% confidence interval [CI]:1.08–1.28, in *intermediate population* areas, and RR = 1.32, 95% CI: 1.20–1.45, in *thinly populated* areas, in 2016). This inverse relationship was found across age, geographical areas, and consecutively over 22 years (1994–2016). In females, no significant difference was detected (RR = 0.96, 95% CI: 0.82–1.13 in *intermediate density* areas and RR = 1.02, 95% CI: 0.85–1.22 in *thinly populated* areas). Hanging was the most common suicide method among males, more frequent in *thinly* (58.8%) than *intermediate* (53.2%) or *densely* (41.4%) *populated* areas.

**Conclusions.:**

A consistent and temporally stable inverse relationship between population density and suicide was found in the male, but not female, population. Men may be more vulnerable to adverse social and economic factors associated with lower population density.

## Introduction

Higher levels of urbanization have been associated with greater risk of psychopathology, including schizophrenia, depression, anxiety, eating disorders, and substance abuse [1,2]. Stress, environmental pollution, easier access to substances of abuse, reduced physical activity and sunlight exposure, and greater genetic risk have all been proposed as possible mediators [3–5]. Mental disorders are associated with increased risk of all-cause mortality and suicide [6]. A number of studies, however, found higher suicide risk in less urbanized areas [7,8].

In the United States, higher suicide rates have been reported in rural areas among the White, American Indian, and Hispanic population, but not among African Americans [7]. The greater availability of firearms in rural areas has been proposed as a mediator of the increased risk [7,9]. Higher rural suicide rates have been reported also in China and Austria [10,11]. A recent meta-analysis of data from four English-speaking countries (Australia, Canada, the United Kingdom, and the United States) found higher suicide rates in rural areas among males, with a widening rural–urban discrepancy [12–15]. Socioeconomic status and social isolation account only in part for this discrepancy in suicide risk [16,17].

However, not all the reports are univocal. No association between urbanization levels and suicide rates was found in the Netherlands [18]. In Denmark, suicide risk was higher in more urbanized areas in 1981–1997, even though, after controlling for socioeconomic and psychiatric variables, it was higher for men in less populated areas [19]. In Scotland, suicide rates were higher in the most and in the least populated quartile as compared with the middle quartiles, with a U-shaped distribution, over the period 1981–1999 [20]. There are also suggestions of a possible historical reversal in the direction of the association between population density and suicide rate with the highest suicide rates in urban areas in the past. For example, in New Zealand, suicide rates were higher in urban than rural areas in 1980–1982, but not any more by the late 1990s, due to increased rates in rural areas [21]. In Italy, an analysis over the years 1969–1984 reported higher suicide rate in urban than nonurban settings, but highly populated suburban settings were considered nonurban settings [22].

Taken together data from the United States, the United Kingdom, Australia, Canada, Austria, and China point to a higher risk of suicide in rural areas, but reports from other countries, such as the Netherlands or Denmark, do not support this association. There are also suggestions that since the mid-1990s the gap rural–urban has become more evident. A better understanding of the distribution of suicide rate by population density may help identify possible risk and protective factors, and lead to more targeted preventive interventions.

We examined the relationship between different degrees of urbanization and suicide incidence in the Italian population over the period 1985–2016. Italy is ethnically, culturally, and socioeconomically less heterogeneous than other countries, such as the United States, with a restrictive regulation of firearms and a universal national health care system that provides medical services to the entire population regardless of income. The study was aimed at addressing the following primary questions: (a) Is there an association between different levels of population density and incidence of suicide? (b) Is there evidence of sex or age effect on such an association? (c) Has the association changed over the last 20 years? and (d) Is the association consistent across the main geographical areas of the country?

## Methods

### Population

The entire Italian population aged 15 years and older (52.4 million in 2016) was examined using the Italian National Institute of Statistics (ISTAT) databases [23]. Italy is divided into 21 administrative regions, including 7,978 local municipalities (in 2016), grouped in four geographical macro-areas: North-West, North-East, Center, and South with Islands. According to the statistical office of the European Union (EUROSTAT), municipalities (also named Local Administrative Units) are classified into three types of areas: *densely populated areas* (also referred to as cities or large urban area), *intermediate density areas* (alternative name: towns and suburbs or small urban area), and *thinly populated areas* (alternative name: rural area) [24]. Specifically, the indicator (DEGURBA) developed by EUROSTAT is based on a criterion of geographical contiguity in combination with the share of local population living in the different type of clusters areas [24]. The classification method has two steps. In a first step, grid cells of 1 km^2^ are classified, according to their population size and density, into one of the following clusters: (a) contiguous grid cells of 1 km^2^ with a density of at least 1,500 inhabitants per km^2^ and a minimum population of 50,000 inhabitants (*high-density cluster/urban center*); (b) clusters of contiguous grid cells of 1 km^2^ with a density of at least 300 inhabitants per km^2^ and a minimum population of 5,000 inhabitants (*urban cluster*); or (c) grid cells outside high-density clusters and urban clusters (*rural grid cells*). In a second step, municipalities are then classified into one of three type of areas: *densely*, *intermediate*, and *thinly populated area. Densely populated* areas have at least 50% of population living in high-density clusters; *intermediate density* areas have less than 50% of the population lives in rural grid cells and less than 50% of population living in high-density clusters; and *thinly populated* areas have more than 50% of the population living in rural grid cells [25,26].

In Italy, about two-thirds (68%) of the municipalities fall into the *thinly populated* group, including an area that corresponds to 72% of the country and is home to 24% of the total population. *Densely populated* areas represent only 3% of the total, occupy about 5% of the Italian territory, and are home to 33% of the Italian population. Municipalities with an *intermediate density* of population represent about 29% of the total municipalities, 23% of the Italian territory, and host 42% of the Italian population [23].

### Mortality data

Data on suicide deaths and overall mortality were obtained from the Italian Mortality Database (IMDB) collected by ISTAT and processed at the Italian National Institute of Health. The IMDB includes all death certificates of Italian residents who died in Italy. The underlying cause of death is coded according to the International Classification of Diseases (ICD). Suicide deaths are coded under “External Causes” with codes E950–E959 (ICD-9 rev.) for the years 1980–2002, and X60–X84, Y87.0 (ICD-10 rev.) for the years 2003–2016 (latest available when the present study was carried out). Data were analyzed at the municipality level grouped into three degree of population density according to EUROSTAT [24–26].

### Data analyses

Age-adjusted mortality rates were computed for the population aged 15 years and over. Rates were standardized (std) with the direct method, using as a reference the Standard European Population [25]. Rate ratios (RRs) and corresponding 95% confidence intervals (CI) were computed using *densely populated areas* as the reference category. Analyses were performed separately for males and females. Rates were expressed per 100,000 individuals per year. Differences between rates were considered to be statistically significant if the corresponding 95% CIs did not overlap.

When the analyses were performed by geographical macro-area, age classes and methods of suicide data were grouped for the period 2010–2016, and rates were computed as annual average. Annual standardized suicide rates (with 95% CI) and RRs (with 95% CI) were computed for each year in the period 1985–2016. Trends of annual standardized suicide rate were analyzed for the period 1985–2016, using joinpoint regression analysis [27]. Estimated annual percentage change (APC) was computed for each detected trend. A maximum of three joinpoints was allowed, and the minimum number “between two joinpoints” and “between a joinpoint and the end of data” was set to two. In the final model, each joinpoint (if any were detected) indicates a significant change in the slope. Joinpoint analysis was applied to the age-adjusted rates (and their standard errors).

Models were estimated, separately for males and females, for level of urbanization. The *p* values for APC were calculated based on a *t* distribution (two-sided test). Where appropriate, the estimated average APC (AAPC) was calculated. The AAPC is a summary measure computed over a fixed interval as a weighted average of the slope coefficients of the joinpoint regression, with weights equal to the length of each detected segment over the interval (obviously, if there is no joinpoint during the selected period, AAPC coincides with APC).

Differences in method used for suicide between the different population density areas were evaluated for the years 2010–2016 combined using the chi-square test (*χ* ^2^) with post-hoc pair-wise comparisons. A two-tailed *p*-value <0.05 was considered statistically significant. The results were interpreted based on post-hoc analysis of the *χ* ^2^ contingency table using the adjusted residual method [28].

Analyses were conducted with IBM-SPSS 25 (IBM-SPSS Corp., Armonk, NY) and SAS 9.4 (SAS Institute, Cary, NC). Rates were computed with an integrated system of programs, PATED (procedure for the spatial analysis of descriptive epidemiology, version 4.3) [29]. Joinpoint regressions were performed by Joinpoint Regression Program, Version 4.7.0.0, by Statistical Research and Applications Branch, National Cancer Institute (https://surveillance.cancer.gov/joinpoint/) [27].

## Results

### Urbanization levels and suicide in 2010–2016

In 2016, among Italian residents aged 15 years and older, there were 3,780 deaths by suicide, 78% of which were males. The standardized suicide rate was 11.60 per 100,000 inhabitants among men and 2.84 among women (Table 1). No statistically significant differences in the all-causes mortality by degree of population density were observed in either males or females (Supplemental Table S1).

**Table 1.** Suicide by sex and population density, age 15 years and older: sdt rates and RRs, Italy, year 2016.

	Population density	*N* of municipalities	*N* of suicides	Population	Std rates per 100,000	RR	95% CI
Males	Densely populated	270	841	8,269,546	10.07	1.00	
	Intermediate density	2,301	1,273	10,707,509	11.79	1.17	(1.08–1.28)
	Thinly populated	5,407	851	6,238,293	13.25	1.32	(1.20–1.45)
	Italy	7,978	2,965	25,215,348	11.60		
Females	Densely populated	270	281	9,230,369	2.82	1.00	
	Intermediate density	2,301	332	11,441,905	2.79	0.96	(0.82–1.13)
	Thinly populated	5,407	202	6,507,656	2.93	1.02	(0.85–1.22)
	Italy	7,978	815	27,179,929	2.84		

Abbreviations: CI, confidence interval; sdt, standardized; RRs, rate ratios.

**Figure 1.**
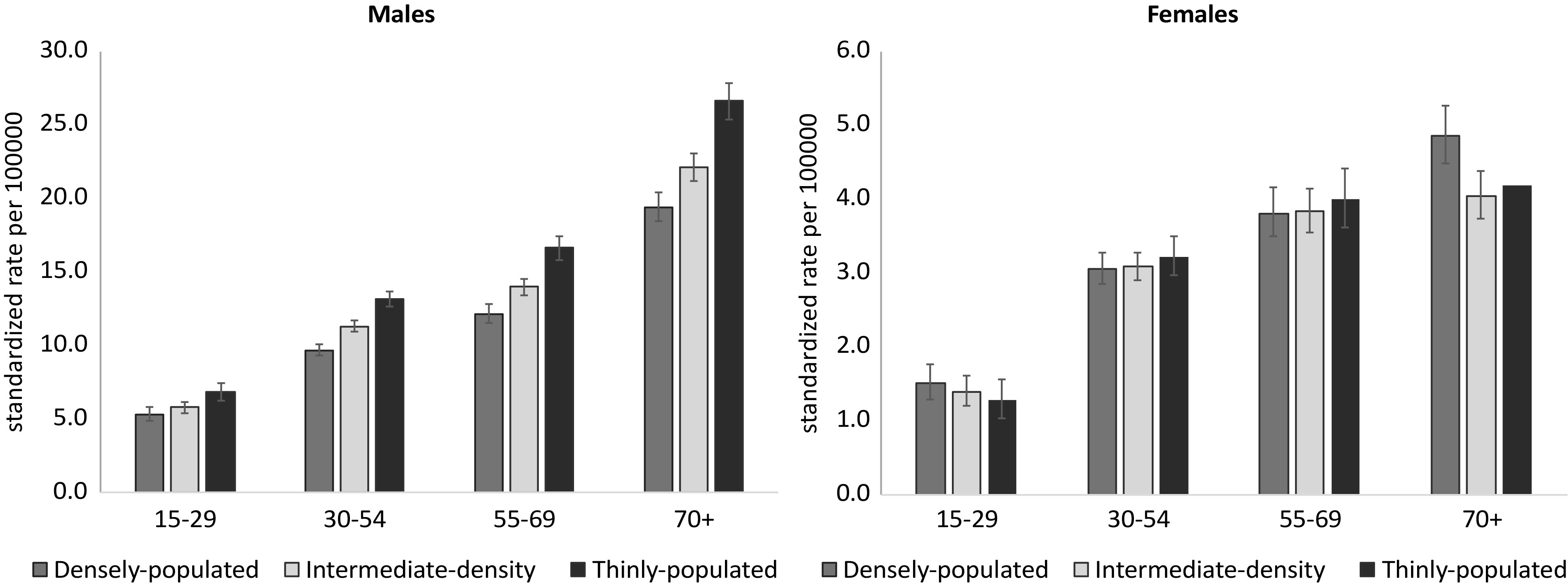
Standardized suicide rates (per 100,000 inhabitants) by population density, age, and sex, Italy, years 2010–2016 (annual average).

Among males living in *densely populated areas*, a suicide rate of 10.07 per 100,000 inhabitants was recorded. Suicide rates were significantly higher in the *thinly populated areas* (13.25 per 100,000; RR = 1.32; 95% CI: 1.20–1.45) and *intermediate populated areas* (11.79 per 100,000; RR = 1.17; 95% CI: 1.08–1.28) compared to the *densely populated areas* (Table 1). Suicide rates were higher in the *thinly populated areas* compared with the *densely populated areas* across the life span. *Intermediate populated areas* had suicide rates overlapping or slightly higher compared to the *densely populated* areas until 30–34 years of age, with differences widening afterward (Figure 1 and Supplemental Figure S1).

Among females, the suicide rate was about 2.84 per 100,000 inhabitants in all the three groups of municipalities, with no statistically significant differences by population density level (Table 1). When examining age, the annual average of suicide rate for the years 2010–2016 did not show significant differences by population density, except among the oldest group (70 years and older), in which higher suicide rates were recorded in *densely populated* areas (Figure 1, Supplemental Figure S1, and Supplemental Table S2).

In the period of observation 2010–2016, for both sexes, the highest suicide rates were recorded in the North-East of Italy (males: 15.21 per 100,000, 95% CI: 14.79–15.64; females: 4.02, 95% CI: 3.82–4.23) and the lowest in the South-Islands (males: 10.82, 95% CI: 10.55–11.09; females: 2.34, 95% CI: 2.22–2.46). Suicide rates were higher in the North-West (13.16, 95% CI: 12.82–13.50 in males and 3.60, 95% CI: 3.44–3.77 in females) and Center (12.29, 95% CI: 11.92–12.68 in males and 2.99, 95% CI: 2.82–3.17 in females) compared to the South-Islands (Supplemental Table S3).

In males, within each macro-region of the country, the highest suicide rates were consistently found in the *thinly populated areas* (Supplemental Figure S2 and Supplemental Table S3). Among females, no consistent association was seen across the geographical macro-regions, with *thinly populated* areas recording higher suicide rates than the *densely populated* areas only in the South, and *thinly urbanized* areas of the North-East showing lower suicide rates than *densely populated areas* (Supplemental Figure S2 and Supplemental Table S3).

### Trends 1985–2016

**Figure 2.**
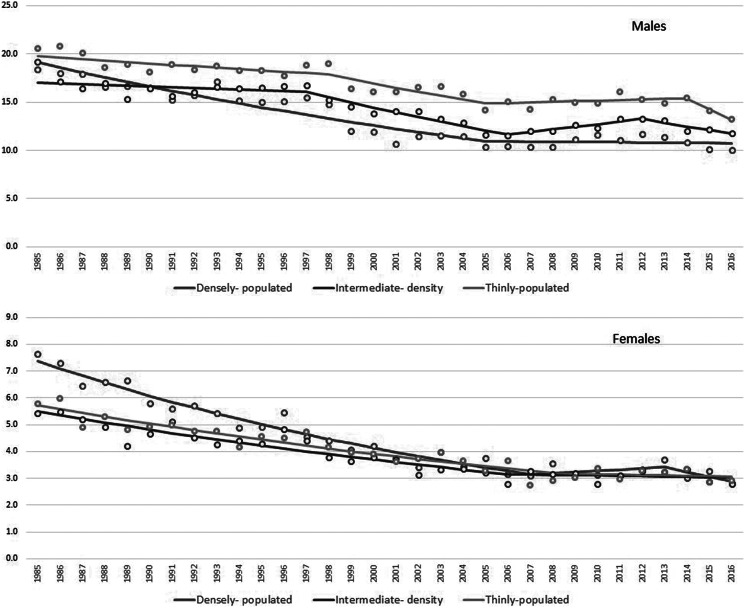
Standardized suicide rates per 100,000 inhabitants (circles) and estimated trends (lines), Italy, years 1985–2016.

Among males, a statistically significant reduction in suicide mortality was observed from 1985 to 2006 (APC 1985–1997: −1.0, *p* < 0.01; APC 1997–2006: −3.1, *p* < 0.01), followed by a nonstatistically significant increase in the years of economic downturn (ACP 2006–2012: +1.6, *p* = 0.10) and a significant decrease in the most recent years (ACP 2012–2016: −3.4, *p* < 0.01). In the *densely* and *thinly populated* areas, there was a statistically significant reduction from 1985 to 2005 (APC 1985–2005: −2.8, *p* < 0.01 in *densely populated* areas; APC 1985–1998: –0.8, APC 1998–2005: −2.6, *p* < 0.01, in *thinly populated* areas). In the *intermediate population density* areas, a significant reduction was observed only from 1997 to 2006 (APC: −3.5, *p* < 0.01) (Figure 2 and Supplemental Table S4). *Thinly populated* areas had the highest suicide rates over the entire period of observation. Until the mid-1990s, suicide rates in *densely* and *intermediate populated* areas mostly overlapped; afterward, *densely populated* areas had the lowest suicide rates (Figure 2, Supplemental Table S5, and Supplemental Figure S3).

When considering the 95% CIs, the suicide rate was statistically significantly lower in the *densely populated* areas than in the *thinly populated* areas in every year from 1994 until 2016 (Supplemental Table S5 and Supplemental Figure S3). During this period, suicide rates in the *intermediate population density* areas were in between those of the *densely* and *thinly populated* areas (Supplemental Figure S3), with statistically significant difference from both of these in the years 2000–2003, 2007–2008, 2011–2013, and 2015. Suicide rates in the *intermediate populated areas* were statistically significantly lower than in the *thinly populated* areas in each year for the period 2000–2015, and higher than in the *densely populated* areas in the years 1999–2003, 2007–2008, 2011–2013, and 2015–2016 (see 95% CI in Supplemental Table S5).

Among females, there was an overall significant reduction in suicide rates from 1985 to 2007 (APC: −3.0, *p* < 0.01). A similar trend was observed in all the three groups of municipalities. Up to the mid-1990s, the *densely populated* areas had the highest suicide rates, while afterward the three population density groups mostly overlapped (Figure 2 and Supplemental Table S5).

### Method of suicide and urbanization level

Among males, hanging was the most frequent method of suicide, accounting for about half of the total suicide deaths (51.5%) (Table 2). The second and third most frequent methods were jumping from a high place (15.6%) and firearms (14.6%). There were statistically significant differences in suicide method by levels of population density (*χ* ^2^ = 965.7, *df* = 18, *p* < 0.01). Hanging was more frequent in the *thinly populated* (58.8%) compared to the *intermediate or densely populated ones* (53.2 and 41.4% respectively). Jumping from high places accounted for 26.2% in the densely populated municipalities, while this method was less frequently used in the *intermediate* (12.7%) and *thinly populated* ones (9.8%). Poisoning by drugs was more frequent in the *densely populated* areas (2.9%). Firearm and drowning suicides were less frequent in the *densely populated* areas but with less marked differences (Table 2 and Supplemental Table S6).

**Table 2:** Percentage distribution of suicide by sex, method, and population density, Italy, years 2010–2016 (annual average).

Males					
Method of suicide		Population density			Total
Definition	ICD-10 code	Densely populated	Intermediate density	Thinly populated	
Hanging	X70	41.4	53.2	58.8	51.5
Firearms	X72–X74	13.3	14.9	15.4	14.6
Fall/jumping from a high place	X80	26.2	12.7	9.8	15.6
Poisoning by carbon monoxide	X67	1.7	2.0	1.8	1.8
Poisoning by drugs	X60–X64	2.9	1.6	1.3	1.9
Poisoning by other substances	X65–<>***X66; X68–X69	1.4	1.5	1.1	1.4
Drowning	X71	2.2	3.2	3.2	2.9
Jumping/lying before moving object	X81	2.8	3.0	1.3	2.4
Cutting and piercing	X78	2.1	2.1	1.7	2.0
Others		6.0	5.7	5.7	5.8
Total	X60–X84, Y87.0	100	100	100	100

Females					
Method of suicide		Population density			Total
Definition	ICD-10 code	Densely populated	Intermediate density	Thinly populated	
Hanging	X70	24.1	35.7	41.8	33.2
Firearms	X72–X74	1.5	2.1	2.9	2.1
Fall/jumping from a high place	X80	48.1	28.5	25.0	34.5
Poisoning by carbon monoxide	X67	0.7	0.9	0.7	0.8
Poisoning by drugs	X60–X64	7.4	6.5	5.6	6.6
Poisoning by other substances	X65–X66; X68–X69	2.9	3.8	3.8	3.5
Drowning	X71	3.9	10.7	9.4	8.0
Jumping/lying before moving object	X81	2.3	3.2	1.7	2.5
Cutting and piercing	X78	1.5	2.0	1.5	1.7
Others		7.5	6.5	7.4	7.0
Total	X60–X84, Y87.0	100	100	100	100

Abbreviation: ICD-10, International Classification of Diseases–10.

Among females, the most frequent method of suicide was jumping from high places (34.5%) followed by hanging (33.2%), drowning (8.0%), and poisoning by drugs (6.6%), with significant differences by the degree of urbanization (*χ* ^2^ = 372.3, *df* = 18, *p* < 0.01). In the *densely populated* municipalities, the most frequent method for suicide was jumping from a high place (48.1%), which was much less common in the *intermediate* (28.5%) and *thinly populated* (25.0%) municipalities. As in males, also in females, hanging was more frequent in *thinly populated* municipalities where it represented 41.8% of suicides as compared to 37.5 and 24.1% in the *intermediate and thinly populated* areas, respectively. Suicide by firearms was more common in *thinly populated* areas than in *densely populated areas* (2.9% vs. 1.5%), whereas drowning was less frequent in *densely populated* areas (3.9 vs. 10.7% in *intermediate* and 9.4% in *thinly* populated areas) (Table 2 and Supplemental Table S6 for statistical significance of post hoc pairwise comparisons).

Differences in suicide methods between the three levels of population density remained statistically significant when controlling by age, in both males (*χ* ^2^ = 1,052.8, *df* = 18, *p* < 0.01) and females (*χ* ^2^ = 355.4, *df* = 18, *p* < 0.01).

## Discussion

These analyses of the entire Italian population data over the period 1985–2016 document an inverse relationship between level of population density of the area of residence and suicide rate in males starting with the mid-1990s. The higher risk for suicide was not limited to rural areas. A gradient of effect was detected, with increasing rates of suicide with decreasing levels of population density. This relationship was seen across age groups and the country macro geographical areas. In females, however, until the mid-1990s, the highest suicide rates were observed in the areas with high population density, with no relationship between population density and suicide rates being detected afterward, except among the oldest age group, who had higher suicide rates if living in areas with high population density.

The results are consistent with previous reports of a higher risk of suicide in rural areas [7,13,14] and support the conclusion that, in males, there is a link between population density and suicide risk. The direction of the association may appear counterintuitive, as urbanization is associated with greater risk for mental illness and substance abuse, which are major risk factors for suicide [1,2]. The higher suicide risk in less populated areas must be explained by factors other than the prevalence of psychiatric illness.

In the United States, the greater availability of firearms in rural areas is considered the main mediator of the greater incidence of suicide [7,9]. Italy has a strict national firearm control regulation. Still firearms may be more prevalent in rural than in urban households because of their use in hunting. In fact, when examining the suicide methods, we found that suicide by firearm was more prevalent in less populated areas, but this was the case also for hanging, which was, by far, the method most commonly used by men across the three population density areas (Table 2). Suicide by jumping from a high place was especially common in more populated areas, possibly reflecting greater availability of this means.

Other important factors that can influence suicide risk are socioeconomic variables, in particular financial stressors and unemployment. However, suicide rate was higher in the more economically prosperous geographical areas of the North-East and North-West of Italy than in the South, where unemployment and poverty are higher [30]. Differences in availability of health services, and especially mental health services, may play a role, but Italy has a universal national health system regardless of income or location [31]. Still, health services may be more scarce or difficult to access in remote rural areas.

The sex difference in the relationship between population density and suicide rate is striking and adds further evidence to the major effect of sex in moderating suicide risk. It suggests that males are especially vulnerable to contextual factors associated with lower population density.

We find intriguing that the association between low population density and higher suicide risk observed among males was consistent over a period of 22 consecutive years of observation (1994–2016), at a time of rise and great expansion of internet-based and wireless means of distant communication, which could have been expected to reduce geographical disparities in health and social variables. It is possible that direct human contact, as distinct from indirect contact through technology, be relevant to psychological health and suicide risk. Opportunities for direct human contact can be expected to be greater in more densely populated areas. It has been reported that that being in the mere presence of other people influences human behavior, even though this may result in a tendency to accept more risk [32]. If a greater opportunity for social contacts influences suicide risk may be worth exploring in future research.

This study has limitations due to the characteristic of the available databases. Besides sex and age, possible confounders related to socioeconomic disparity, health services, or access to firearms were not controlled for. No information was available on psychiatric status of those who died of suicide or on the role of possible mediators, such as alcohol abuse, unemployment, marital status, level of education, disabilities, or health services. Despite the possibility of underestimating the real incidence of suicide when relying on population national reporting systems, such as ISTAT, previous analyses found little evidence of systematic biases in reporting [33].

These results have implications for further research and the development of more targeted preventive interventions. The possible mediators of the inverse relationship between population density and suicide risk should be further investigated. While greater availability of firearms, like in the United States, or agricultural toxic products, like in other countries, may account for higher suicide risk in rural areas[34], this does not seem the case in our study. The data from the Italian population suggest that factors other than availability of lethal suicide methods are at play. These might be related to differences in social contacts characteristics in areas of different population density. A better understanding of the characteristics of an individual’s social relationships with respect to population density may be relevant to evaluating suicide risk [35].

In conclusion, a gradient of lowering risk of suicide with increasing levels of population density was found in the male, but not female, population. The finding, sustained over the past 20 years, deserves further investigation as to possible mediators.

## Data Availability

The data that support the findings of this study are available from the Italian National Institute of Statistics (www.istat.it), and the analytical methods are available from the authors upon request.

## Supplementary material

For supplementary material accompanying this paper visit http://dx.doi.org/10.1192/j.eurpsy.2020.69. click here to view supplementary material
