# Supplementary material for: Does population density moderate suicide risk? An Italian population study over the last 30 years
Source: Eur Psychiatry. 2020 Jul 1;63(1):e70. doi: 10.1192/j.eurpsy.2020.69 (PMC7443791; doi:10.1192/j.eurpsy.2020.69)
Supplement: Supplementary file 1 [file S0924933820000693sup001.zip › S0924933820000693supp004.docx]

**Supplemental Table 1 - Overall mortality by population density for males and females, age 15 years and over. Standardized (Std) rates and Rate Ratios (RR) with corresponding 95% Confidence Intervals (CI). Italy, year 2016**

|  | Population density | | N of municipalities | N of deaths | Population | Std rates per 100,000 | RR | 95% CI |
| --- | --- | --- | --- | --- | --- | --- | --- | --- |
| Males | Densely populated | | 270 | 95,692 | 8,269,546 | 1,238 | 1.00 |  |
|  | Intermediate-density | | 2301 | 118,409 | 10,707,509 | 1,247 | 0.99 | (0.95-1.03) |
|  | Thinly populated | | 5407 | 80,016 | 6,238,293 | 1,276 | 1.04 | (1.00-1.08) |
|  | *ITALY* | | *7978* | *294,117* | *25,215,348* | *11,251* |  |  |
| Females | Densely populated | | 270 | 108,638 | 9,230,369 | 812 | 1.00 |  |
|  | Intermediate-density | 2301 | | 125,704 | 11,441,905 | 808 | 0.98 | (0.96-1.01) |
|  | Thinly populated | 5407 | | 83,786 | 6,507,656 | 824 | 1.01 | (0.98-1.03) |
|  | *ITALY* | *7978* | | *318,128* | *27,179,930* | 813 |  |  |

**Supplemental Table 2 –Suicide by population density, age, and sex. Standardized (Std) rates and Rate Ratios (RR) with corresponding 95% Confidence Intervals (95% CI). Italy, years 2010-2016 (annual average)**

| Age | Population density | |  | Suicide number  2010-2016 | Std rates per 100,000 | RR  (95%CI) | Suicide n.  2010-2016 | Std rates per 100,000 | RR  (95%CI) |  |
| --- | --- | --- | --- | --- | --- | --- | --- | --- | --- | --- |
|  |  | |  | Males | Males | Males | Females | Females | Females |  |
| 15-29 years | Densely-populated | |  | 573 | 5.32 | 1.00 | 157 | 1.51 | 1.00 |  |
|  | Intermediate-density | |  | 817 | 5.80 | 1.09 | 190 | 1.40 | 0.92 |  |
|  |  | |  |  |  | (0.89-1.35) |  |  | (0.78-1.08) |  |
|  | Thinly-populated | |  | 557 | 6.84 | 1.29* | 99 | 1.29 | 0.84 |  |
|  |  | |  |  |  | (1.05-1.58) |  |  | (0.69-1.03) |  |
| *Overall 15-29 years* | | |  | *1948* | *5.92* |  | *446* | *1.41* |  |  |
| 30-54 years | Densely-populated | |  | 2435 | 9.70 | 1.00 | 810 | 3.06 | 1.00 |  |
|  | Intermediate-density | |  | 3776 | 11.32 | 1.17* | 1042 | 3.09 | 1.01 |  |
|  |  | |  |  |  | (1.11-1.23) |  |  | (0.92-1.11) |  |
|  | Thinly-populated | |  | 2496 | 13.15 | 1.36* | 603 | 3.23 | 1.06 |  |
|  |  | |  |  |  | (1.29-1.44) |  |  | (0.95-1.17) |  |
| *Overall 30-54 years* | | |  | *8708* | *11.24* |  | *2455* | *3.11* |  |  |
| 55-69 years | Densely-populated | |  | 1436 | 12.16 | 1.00 | 516 | 3.81 | 1.00 |  |
|  | Intermediate-density | |  | 2175 | 13.99 | 1.15* | 638 | 3.84 | 1.01 |  |
|  |  | |  |  |  | (1.03-1.29) |  |  | (0.92-1.09) |  |
|  | Thinly-populated | |  | 1570 | 16.65 | 1.37* | 382 | 4.00 | 1.05 |  |
|  |  | |  |  |  | (1.22-1.54) |  |  | (0.95-1.15) |  |
| *Overall 55-69 years* | | |  | *5181* | *14.08* |  | *1538* | *3.88* |  |  |
| 70 years and over | Densely-populated | |  | 1684 | 19.44 | 1.00 | 653 | 4.87 | 1.00 |  |
|  | Intermediate-density | |  | 2360 | 22.16 | 1.14* | 624 | 4.05 | 0.83* |  |
|  |  | |  |  |  | (1.09-1.20) |  |  | (0.75-0.93) |  |
|  | Thinly-populated | |  | 1918 | 26.68 | 1.39* | 410 | 4.19 | 0.85* |  |
|  |  | |  |  |  | (1.32-1.46) |  |  | (0.76-0.97) |  |
| *Overall 70 years and over* | | |  | *5963* | *22.36* |  | *1687* | *4.36* |  |  |
|  | |  |  |  |  |  |  |  |  |  |

**Supplemental Table 3 –Suicide by population density and geographical macro area. Standardized (Std) rates and Rate Ratios (RR) with corresponding 95% Confidence Intervals (95% CI). Italy, years 2010-2016 (annual average). Males and Females**

|  | Population density | N of municipalities | Suicide n.  2010-2016 | Std rates per 100,000 | RR  (95%CI) | Suicide n.  2010-2016 | Std rates per 100,000 | RR  (95%CI) |  |
| --- | --- | --- | --- | --- | --- | --- | --- | --- | --- |
|  |  |  | Males | Males | Males | Females | Females | Females |  |
| North - West | Densely-populated | 133 | 1,933 | 11.40 | 1.00 | 722 | 3.59 | 1.00 |  |
|  | Intermediate-density | 1029 | 2,781 | 12.95 | 1.15* | 833 | 3.50 | 0.96 |  |
|  |  |  |  |  | (1.08-1.22) |  |  | (0.87-1.06) |  |
|  | Thinly-populated | 1872 | 1,430 | 17.28 | 1.53* | 340 | 3.88 | 1.07 |  |
|  |  |  |  |  | (1.43-1.64) |  |  | (0.94-1.21) |  |
| *Overall North West* | | *3034* | *144* | *13.16* |  | *1895* | *3.60* |  |  |
| North - East | Densely-populated | 19 | 1,283 | 14.36 | 1.00 | 470 | 4.42 | 1.00 |  |
|  | Intermediate-density | 455 | 2,321 | 15.10 | 1.06 | 673 | 3.91 | 0.89 |  |
|  |  |  |  |  | (0.99-1.13) |  |  | (0.79-1.00) |  |
|  | Thinly-populated | 943 | 1,542 | 16.21 | 1.14* | 380 | 3.78 | 0.84* |  |
|  |  |  |  |  | (1.06-1.23) |  |  | (0.74-0.97) |  |
| *Overall North-East* | | *1417* | *5146* | *15.21* |  | *1523* | *4.02* |  |  |
| Centre | Densely-populated | 13 | 1,388 | 11.27 | 1.00 | 477 | 3.18 | 1.00 |  |
|  | Intermediate-density | 224 | 1,537 | 11.92 | 1.06 | 408 | 2.77 | 0.86* |  |
|  |  |  |  |  | (0.98-1.14) |  |  | (0.76-0.99) |  |
|  | Thinly-populated | 738 | 1,300 | 14.24 | 1.27* | 301 | 3.03 | 0.95 |  |
|  |  |  |  |  | (1.18-1.37) |  |  | (0.82-1.10) |  |
| *Overall Centre* | | *975* | *4225* | *12.29* |  | *1186* | *2.99* |  |  |
| South and Islands | Densely-populated | 105 | 1,524 | 8.50 | 1.00 | 467 | 2.25 | 1.00 |  |
|  | Intermediate-density | 593 | 2,489 | 10.74 | 1.26* | 580 | 2.26 | 1.01 |  |
|  |  |  |  |  | (1.18-1.34) |  |  | (0.90-1.12) |  |
|  | Thinly-populated | 1854 | 2,269 | 13.19 | 1.54* | 473 | 2.53 | 1.13* |  |
|  |  |  |  |  | (1.44-1.64) |  |  | (1.01-1.27) |  |
| *Overall South-Islands* | | *2552* | *6282* | *10.82* |  | *1520* | *2.34* |  |  |
| Italy | Densely-populated | 270 | 6,128 | 10.94 | 1.00 | 2,136 | 3.20 | 1.00 |  |
|  | Intermediate-density | 2301 | 9,128 | 12.55 | 1.15* | 2,494 | 3.06 | 0.95 |  |
|  |  |  |  |  | (1.11-1.19) |  |  | (0.90-1.01) |  |
|  | Thinly-populated | 5407 | 6,541 | 14.84 | 1.36* | 1,494 | 3.15 | 0.98 |  |
|  |  |  |  |  | (1.32-1.41) |  |  | (0.91-1.04) |  |
| *Overall Italy* | | *7978* | *21797* | *12.61* |  | *6124* | *3.13* |  |  |

**Supplemental Table 4 - Suicide by level of population density: estimated Annual Percentage Change (APC) and Average Annual Percentage Change (AAPC) and corresponding 95% confidence intervals (95% CI), Italy, 1985–2016.**

1. **Males**

| Cohort  Population density | Segment | Lower Endpoint | Upper Endpoint | APC | Lower 95%CI | Upper 95%CI | Test Statistic (t) | Prob > \|t\| | |
| --- | --- | --- | --- | --- | --- | --- | --- | --- | --- |
| Densely populated | 1 | 1985 | 2005 | -2.8* | -3.3 | -2.2 | -10.7 | <0.01 |  |
|  | 2 | 2005 | 2016 | -0.2 | -1.4 | +1.1 | -0.3 | 0.80 |  |
|  | *AAPC* | 1985 | 2016 | -1.8* | -2.4 | -1.3 | -6.7 | <0.01 |  |
| Intermediate-density | 1 | 1985 | 1997 | -0.4 | -1.2 | +0.3 | -1.3 | 0.20 |  |
|  | 2 | 1997 | 2006 | -3.5* | -4.7 | -2.3 | -5.9 | <0.01 |  |
|  | 3 | 2006 | 2012 | +2.2 | -0.3 | +4.8 | +1.8 | 0.10 |  |
|  | 4 | 2012 | 2016 | -3.1 | -6.2 | +0.2 | -2.0 | 0.10 |  |
|  | *AAPC* | 1985 | 2016 | -1.2* | -1.9 | -0.4 | -3.1 | <0.01 |  |
| Thinly-populated | 1 | 1985 | 1998 | -0.8* | -1.3 | -0.2 | -3.1 | <0.01 |  |
|  | 2 | 1998 | 2005 | -2.6* | -4.0 | -1.2 | -3.8 | <0.01 |  |
|  |  | 2005 | 2014 | +0.4 | -0.5 | +1.3 | +0.9 | 0.40 |  |
|  | 3 | 2014 | 2016 | -7.4 | -15.1 | +1.0 | -1.9 | 0.10 |  |
|  | *AAPC* | 1985 | 2016 | -1.3* | -2.0 | -0.6 | -3.7 | <0.01 |  |
| Overall (Italy) | 1 | 1985 | 1997 | -1.0* | -1.6 | -0.3 | -3 | <0.01 |  |
|  | 2 | 1997 | 2006 | -3.1* | -4.2 | -2.0 | -5.8 | <0.01 |  |
|  | 3 | 2006 | 2012 | +1.6 | -0.5 | +3.8 | 1.6 | 0.10 |  |
|  | 4 | 2012 | 2016 | -3.4* | -6.1 | -0.6 | -2.5 | <0.01 |  |
|  | *AAPC* | *1985* | *2016* | *-1.4** | *-2.1* | *-0.8* | *-4.3* | *<0.01* |  |

1. **Females**

| Cohort  Population density | Segment | Lower Endpoint | Upper Endpoint | APC | Lower 95%CI | Upper 95% CI | Test Statistic (t) | Prob > \|t\| |  |
| --- | --- | --- | --- | --- | --- | --- | --- | --- | --- |
| Densely populated | 1 | 1985 | 2007 | -3.8* | -4.2 | -3.4 | -19.7 | <0.01 |  |
|  | 2 | 2007 | 2013 | +1.4 | -3.0 | +6.0 | +0.7 | 0.50 |  |
|  | 3 | 2013 | 2016 | -5.5 | -14.8 | +4.9 | -1.1 | 0.30 |  |
|  | *AAPC* | 1985 | 2016 | -3.0* | -4.2 | -1.7 | -4.6 | <0.01 |  |
| Intermediate-density | 1 | 1985 | 2006 | -2.6* | -3.1 | -2.1 | -10.8 | <0.01 |  |
|  | 2 | 2006 | 2016 | -0.4 | -2.0 | +1.2 | -0.5 | 0.60 |  |
|  | *AAPC* | 1985 | 2016 | -1.9* | -2.5 | -1.3 | -6.4 | <0.01 |  |
| Thinly-populated | 1 | 1985 | 2008 | -2.5* | -2.9 | -2.1 | -12.4 | <0.01 |  |
|  | 2 | 2008 | 2016 | -0.5 | -2.8 | +1.8 | -0.4 | 0.70 |  |
|  | *AAPC* | 1985 | 2016 | -2.0* | -2.6 | -1.4 | -6.1 | <0.01 |  |
| Overall (Italy) | 1 | 1985 | 2007 | -3.0* | -3.3 | -2.7 | -20.3 | <0.01 |  |
|  | 2 | 2007 | 2016 | -0.4 | -1.7 | 0.9 | -0.6 | 0.60 |  |
|  | *AAPC* | *1985* | *2016* | *-2.2** | *-2.6* | *-1.8* | *-10.5* | *<0.01* |  |

**Supplemental Table 5 - Suicide by population density of area of residence and sex. Number of suicides (n), standardized (std) rate, and Rate Ratios (RR) with corresponding 95% Confidence Intervals (95% CI). Italy, years 1985-2016**

**MALES**

|  | **Population density** | | | | | | | | | | | | | | | | | | | | | | | | | | | | | | | | | |
| --- | --- | --- | --- | --- | --- | --- | --- | --- | --- | --- | --- | --- | --- | --- | --- | --- | --- | --- | --- | --- | --- | --- | --- | --- | --- | --- | --- | --- | --- | --- | --- | --- | --- | --- |
|  | **Densely populated** | | | | | | | | **Intermediate density** | | | | | | | | | | | |  | **Thinly populated** | | | | | | | | | | | |  |
| **Year** | **n** | **Std rate** | **95%CI** | | | | | **RR** | **n** | **Std rate** | **95%CI** | | | | | **RR** | **95%CI** | | | | | **n** | **Std rate** | **95%CI** | | | | | **RR** | **95%CI** | | | | |
| **1985** | 1130 | 18.4 | ( | 17.2 | - | 19.9 | ) | 1.00 | 1239 | 19.2 | ( | 17.7 | - | 21.1 | ) | 1.04 | ( | 0.96 | - | 1.12 | ) | 950 | 20.5 | ( | 18.1 | - | 25.2 | ) | 1.13 | ( | 1.03 | - | 1.23 | ) |
| **1986** | 1108 | 18.0 | ( | 16.7 | - | 19.5 | ) | 1.00 | 1154 | 17.1 | ( | 15.9 | - | 18.8 | ) | 0.96 | ( | 0.87 | - | 1.06 | ) | 980 | 20.8 | ( | 18.6 | - | 24.6 | ) | 1.18 | ( | 1.06 | - | 1.31 | ) |
| **1987** | 1099 | 17.9 | ( | 16.6 | - | 19.5 | ) | 1.00 | 1134 | 16.4 | ( | 15.3 | - | 18.0 | ) | 0.96 | ( | 0.88 | - | 1.04 | ) | 984 | 20.1 | ( | 18.8 | - | 22.9 | ) | 1.21 | ( | 1.11 | - | 1.31 | ) |
| **1988** | 1004 | 16.6 | ( | 15.3 | - | 18.1 | ) | 1.00 | 1148 | 17.0 | ( | 15.8 | - | 18.5 | ) | 1.06 | ( | 0.97 | - | 1.15 | ) | 906 | 18.6 | ( | 17.3 | - | 21.0 | ) | 1.21 | ( | 1.11 | - | 1.33 | ) |
| **1989** | 1087 | 16.7 | ( | 15.6 | - | 18.0 | ) | 1.00 | 1114 | 15.3 | ( | 14.3 | - | 16.6 | ) | 0.94 | ( | 0.86 | - | 1.02 | ) | 887 | 18.9 | ( | 17.2 | - | 21.5 | ) | 1.10 | ( | 1.01 | - | 1.21 | ) |
| **1990** | 1069 | 16.5 | ( | 15.4 | - | 17.8 | ) | 1.00 | 1164 | 16.4 | ( | 15.3 | - | 17.7 | ) | 0.99 | ( | 0.91 | - | 1.07 | ) | 908 | 18.2 | ( | 16.9 | - | 20.0 | ) | 1.13 | ( | 1.04 | - | 1.24 | ) |
| **1991** | 1004 | 15.3 | ( | 14.2 | - | 16.5 | ) | 1.00 | 1183 | 15.7 | ( | 14.7 | - | 16.8 | ) | 1.06 | ( | 0.97 | - | 1.15 | ) | 976 | 18.9 | ( | 17.7 | - | 20.4 | ) | 1.29 | ( | 1.18 | - | 1.41 | ) |
| **1992** | 1059 | 15.7 | ( | 14.6 | - | 16.9 | ) | 1.00 | 1248 | 16.0 | ( | 15.1 | - | 17.1 | ) | 1.05 | ( | 0.96 | - | 1.14 | ) | 969 | 18.4 | ( | 17.2 | - | 19.7 | ) | 1.20 | ( | 1.10 | - | 1.31 | ) |
| **1993** | 1136 | 17.2 | ( | 16.0 | - | 18.5 | ) | 1.00 | 1307 | 16.6 | ( | 15.6 | - | 17.7 | ) | 1.01 | ( | 0.94 | - | 1.10 | ) | 999 | 18.8 | ( | 17.6 | - | 20.1 | ) | 1.15 | ( | 1.06 | - | 1.25 | ) |
| **1994** | 1052 | 15.2 | ( | 14.1 | - | 16.3 | ) | 1.00 | 1298 | 16.4 | ( | 15.5 | - | 17.5 | ) | 1.08 | ( | 0.99 | - | 1.17 | ) | 993 | 18.3 | ( | 17.1 | - | 19.6 | ) | 1.23 | ( | 1.13 | - | 1.34 | ) |
| **1995** | 1064 | 15.0 | ( | 14.0 | - | 16.1 | ) | 1.00 | 1320 | 16.5 | ( | 15.5 | - | 17.6 | ) | 1.07 | ( | 0.99 | - | 1.17 | ) | 987 | 18.3 | ( | 17.1 | - | 19.6 | ) | 1.21 | ( | 1.11 | - | 1.31 | ) |
| **1996** | 1046 | 15.1 | ( | 14.1 | - | 16.2 | ) | 1.00 | 1373 | 16.7 | ( | 15.7 | - | 17.8 | ) | 1.13 | ( | 1.04 | - | 1.22 | ) | 986 | 17.7 | ( | 16.6 | - | 19.0 | ) | 1.23 | ( | 1.13 | - | 1.34 | ) |
| **1997** | 1080 | 15.5 | ( | 14.5 | - | 16.6 | ) | 1.00 | 1398 | 16.7 | ( | 15.8 | - | 17.8 | ) | 1.10 | ( | 1.02 | - | 1.20 | ) | 1031 | 18.8 | ( | 17.6 | - | 20.1 | ) | 1.24 | ( | 1.14 | - | 1.35 | ) |
| **1998** | 1033 | 14.7 | ( | 13.8 | - | 15.8 | ) | 1.00 | 1269 | 15.2 | ( | 14.3 | - | 16.2 | ) | 1.04 | ( | 0.94 | - | 1.14 | ) | 1055 | 19.0 | ( | 17.8 | - | 20.2 | ) | 1.30 | ( | 1.17 | - | 1.44 | ) |
| **1999** | 853 | 12.0 | ( | 11.1 | - | 13.0 | ) | 1.00 | 1232 | 14.5 | ( | 13.7 | - | 15.5 | ) | 1.21 | ( | 1.11 | - | 1.32 | ) | 917 | 16.4 | ( | 15.3 | - | 17.5 | ) | 1.38 | ( | 1.25 | - | 1.51 | ) |
| **2000** | 846 | 11.9 | ( | 11.1 | - | 12.9 | ) | 1.00 | 1196 | 13.8 | ( | 13.0 | - | 14.7 | ) | 1.17 | ( | 1.07 | - | 1.28 | ) | 900 | 16.1 | ( | 15.0 | - | 17.2 | ) | 1.36 | ( | 1.24 | - | 1.49 | ) |
| **2001** | 773 | 10.7 | ( | 9.9 | - | 11.6 | ) | 1.00 | 1225 | 14.1 | ( | 13.3 | - | 15.0 | ) | 1.30 | ( | 1.19 | - | 1.43 | ) | 906 | 16.1 | ( | 15.0 | - | 17.2 | ) | 1.50 | ( | 1.36 | - | 1.65 | ) |
| **2002** | 830 | 11.5 | ( | 10.7 | - | 12.4 | ) | 1.00 | 1240 | 14.0 | ( | 13.2 | - | 14.9 | ) | 1.22 | ( | 1.12 | - | 1.33 | ) | 942 | 16.5 | ( | 15.5 | - | 17.7 | ) | 1.45 | ( | 1.32 | - | 1.59 | ) |
| **2003** | 847 | 11.5 | ( | 10.7 | - | 12.4 | ) | 1.00 | 1155 | 13.3 | ( | 12.5 | - | 14.1 | ) | 1.11 | ( | 1.01 | - | 1.21 | ) | 948 | 16.6 | ( | 15.5 | - | 17.7 | ) | 1.42 | ( | 1.29 | - | 1.56 | ) |
| **2004** | 831 | 11.5 | ( | 10.7 | - | 12.4 | ) | 1.00 | 1168 | 12.9 | ( | 12.1 | - | 13.7 | ) | 1.13 | ( | 1.03 | - | 1.24 | ) | 915 | 15.8 | ( | 14.8 | - | 16.9 | ) | 1.39 | ( | 1.27 | - | 1.53 | ) |
| **2005** | 766 | 10.4 | ( | 9.6 | - | 11.2 | ) | 1.00 | 1078 | 11.6 | ( | 10.9 | - | 12.4 | ) | 1.12 | ( | 1.04 | - | 1.21 | ) | 838 | 14.2 | ( | 13.2 | - | 15.2 | ) | 1.38 | ( | 1.27 | - | 1.50 | ) |
| **2006** | 774 | 10.4 | ( | 9.7 | - | 11.2 | ) | 1.00 | 1076 | 11.5 | ( | 10.8 | - | 12.3 | ) | 1.10 | ( | 1.00 | - | 1.21 | ) | 881 | 15.0 | ( | 14.0 | - | 16.1 | ) | 1.43 | ( | 1.30 | - | 1.58 | ) |
| **2007** | 777 | 10.4 | ( | 9.6 | - | 11.2 | ) | 1.00 | 1136 | 12.0 | ( | 11.3 | - | 12.7 | ) | 1.15 | ( | 1.05 | - | 1.26 | ) | 846 | 14.2 | ( | 13.3 | - | 15.3 | ) | 1.36 | ( | 1.24 | - | 1.50 | ) |
| **2008** | 788 | 10.3 | ( | 9.6 | - | 11.1 | ) | 1.00 | 1158 | 12.0 | ( | 11.3 | - | 12.7 | ) | 1.14 | ( | 1.04 | - | 1.25 | ) | 919 | 15.3 | ( | 14.3 | - | 16.4 | ) | 1.46 | ( | 1.33 | - | 1.60 | ) |
| **2009** | 849 | 11.1 | ( | 10.4 | - | 12.0 | ) | 1.00 | 1247 | 12.7 | ( | 11.9 | - | 13.4 | ) | 1.14 | ( | 1.04 | - | 1.24 | ) | 914 | 14.9 | ( | 14.0 | - | 16.0 | ) | 1.35 | ( | 1.23 | - | 1.48 | ) |
| **2010** | 885 | 11.6 | ( | 10.8 | - | 12.4 | ) | 1.00 | 1229 | 12.3 | ( | 11.6 | - | 13.1 | ) | 1.07 | ( | 0.99 | - | 1.15 | ) | 913 | 14.9 | ( | 13.9 | - | 15.9 | ) | 1.29 | ( | 1.20 | - | 1.40 | ) |
| **2011** | 851 | 11.0 | ( | 10.3 | - | 11.8 | ) | 1.00 | 1334 | 13.2 | ( | 12.5 | - | 14.0 | ) | 1.20 | ( | 1.10 | - | 1.31 | ) | 997 | 16.1 | ( | 15.1 | - | 17.1 | ) | 1.47 | ( | 1.34 | - | 1.61 | ) |
| **2012** | 917 | 11.7 | ( | 10.9 | - | 12.5 | ) | 1.00 | 1355 | 13.3 | ( | 12.5 | - | 14.0 | ) | 1.13 | ( | 1.04 | - | 1.23 | ) | 955 | 15.3 | ( | 14.3 | - | 16.3 | ) | 1.31 | ( | 1.20 | - | 1.43 | ) |
| **2013** | 909 | 11.4 | ( | 10.6 | - | 12.2 | ) | 1.00 | 1376 | 13.1 | ( | 12.4 | - | 13.9 | ) | 1.17 | ( | 1.07 | - | 1.27 | ) | 940 | 14.9 | ( | 14.0 | - | 15.9 | ) | 1.32 | ( | 1.21 | - | 1.45 | ) |
| **2014** | 886 | 10.8 | ( | 10.1 | - | 11.6 | ) | 1.00 | 1264 | 12.0 | ( | 11.4 | - | 12.8 | ) | 1.11 | ( | 1.03 | - | 1.19 | ) | 982 | 15.5 | ( | 14.5 | - | 16.5 | ) | 1.43 | ( | 1.33 | - | 1.55 | ) |
| **2015** | 839 | 10.1 | ( | 9.4 | - | 10.8 | ) | 1.00 | 1297 | 12.2 | ( | 11.5 | - | 12.9 | ) | 1.20 | ( | 1.11 | - | 1.30 | ) | 903 | 14.1 | ( | 13.2 | - | 15.1 | ) | 1.40 | ( | 1.28 | - | 1.52 | ) |
| **2016** | 841 | 10.1 | ( | 9.4 | - | 10.8 | ) | 1.00 | 1273 | 11.8 | ( | 11.1 | - | 12.5 | ) | 1.17 | ( | 1.08 | - | 1.28 | ) | 851 | 13.2 | ( | 12.4 | - | 14.2 | ) | 1.32 | ( | 1.20 | - | 1.45 | ) |

**FEMALES**

| **Population density** | | | | | | | | | | | | | | | | | | | | | | | | | | | | | | | | | | |
| --- | --- | --- | --- | --- | --- | --- | --- | --- | --- | --- | --- | --- | --- | --- | --- | --- | --- | --- | --- | --- | --- | --- | --- | --- | --- | --- | --- | --- | --- | --- | --- | --- | --- | --- |
|  | **Densely populated** | | | | | | | | **Intermediate density** | | | | | | | | | | | |  | **Thinly populated** | | | | | | | | | | | |  |
| **Year** | **n** | **Std rate** | **95%CI** | | | | | **RR** | **n** | **Std rate** | **95%CI** | | | | | **RR** | **95%CI** | | | | | **n** | **Std rate** | **95%CI** | | | | | **RR** | **95%CI** | | | | |
| **1985** | 614 | 7.6 | ( | 7.0 | - | 8.3 | ) | 1.00 | 437 | 5.4 | ( | 4.9 | - | 6.0 | ) | 0.71 | ( | 0.63 | - | 0.81 | ) | 324 | 5.8 | ( | 5.2 | - | 6.6 | ) | 0.78 | ( | 0.68 | - | 0.89 | ) |
| **1986** | 599 | 7.3 | ( | 6.7 | - | 7.9 | ) | 1.00 | 450 | 5.5 | ( | 5.0 | - | 6.1 | ) | 0.75 | ( | 0.66 | - | 0.84 | ) | 333 | 6.0 | ( | 5.3 | - | 6.8 | ) | 0.82 | ( | 0.72 | - | 0.94 | ) |
| **1987** | 531 | 6.5 | ( | 5.9 | - | 7.1 | ) | 1.00 | 436 | 5.2 | ( | 4.7 | - | 5.8 | ) | 0.81 | ( | 0.71 | - | 0.92 | ) | 275 | 4.9 | ( | 4.3 | - | 5.6 | ) | 0.76 | ( | 0.66 | - | 0.88 | ) |
| **1988** | 551 | 6.6 | ( | 6.0 | - | 7.2 | ) | 1.00 | 422 | 4.9 | ( | 4.5 | - | 5.5 | ) | 0.75 | ( | 0.66 | - | 0.85 | ) | 302 | 5.3 | ( | 4.7 | - | 6.1 | ) | 0.81 | ( | 0.70 | - | 0.93 | ) |
| **1989** | 564 | 6.7 | ( | 6.1 | - | 7.3 | ) | 1.00 | 369 | 4.2 | ( | 3.8 | - | 4.7 | ) | 0.63 | ( | 0.55 | - | 0.72 | ) | 274 | 4.8 | ( | 4.3 | - | 5.5 | ) | 0.72 | ( | 0.62 | - | 0.83 | ) |
| **1990** | 500 | 5.8 | ( | 5.3 | - | 6.4 | ) | 1.00 | 419 | 4.7 | ( | 4.2 | - | 5.2 | ) | 0.80 | ( | 0.70 | - | 0.91 | ) | 282 | 4.9 | ( | 4.4 | - | 5.6 | ) | 0.83 | ( | 0.72 | - | 0.96 | ) |
| **1991** | 482 | 5.6 | ( | 5.1 | - | 6.1 | ) | 1.00 | 459 | 5.1 | ( | 4.7 | - | 5.7 | ) | 0.90 | ( | 0.81 | - | 1.00 | ) | 297 | 5.0 | ( | 4.4 | - | 5.6 | ) | 0.89 | ( | 0.79 | - | 1.01 | ) |
| **1992** | 491 | 5.7 | ( | 5.2 | - | 6.3 | ) | 1.00 | 414 | 4.5 | ( | 4.1 | - | 5.0 | ) | 0.79 | ( | 0.71 | - | 0.89 | ) | 287 | 4.8 | ( | 4.2 | - | 5.4 | ) | 0.84 | ( | 0.74 | - | 0.95 | ) |
| **1993** | 468 | 5.4 | ( | 4.9 | - | 6.0 | ) | 1.00 | 397 | 4.3 | ( | 3.9 | - | 4.8 | ) | 0.79 | ( | 0.72 | - | 0.88 | ) | 289 | 4.7 | ( | 4.2 | - | 5.4 | ) | 0.88 | ( | 0.79 | - | 0.98 | ) |
| **1994** | 426 | 4.9 | ( | 4.4 | - | 5.4 | ) | 1.00 | 413 | 4.4 | ( | 4.0 | - | 4.9 | ) | 0.89 | ( | 0.78 | - | 1.02 | ) | 254 | 4.2 | ( | 3.7 | - | 4.7 | ) | 0.85 | ( | 0.73 | - | 0.99 | ) |
| **1995** | 430 | 4.9 | ( | 4.5 | - | 5.4 | ) | 1.00 | 412 | 4.3 | ( | 3.9 | - | 4.8 | ) | 0.88 | ( | 0.77 | - | 1.01 | ) | 283 | 4.6 | ( | 4.0 | - | 5.2 | ) | 0.94 | ( | 0.81 | - | 1.09 | ) |
| **1996** | 479 | 5.5 | ( | 5.0 | - | 6.0 | ) | 1.00 | 457 | 4.8 | ( | 4.4 | - | 5.3 | ) | 0.87 | ( | 0.76 | - | 0.99 | ) | 277 | 4.5 | ( | 4.0 | - | 5.1 | ) | 0.82 | ( | 0.71 | - | 0.95 | ) |
| **1997** | 404 | 4.6 | ( | 4.1 | - | 5.0 | ) | 1.00 | 434 | 4.4 | ( | 4.0 | - | 4.9 | ) | 0.96 | ( | 0.84 | - | 1.10 | ) | 296 | 4.7 | ( | 4.2 | - | 5.3 | ) | 1.03 | ( | 0.89 | - | 1.20 | ) |
| **1998** | 388 | 4.4 | ( | 4.0 | - | 4.9 | ) | 1.00 | 374 | 3.8 | ( | 3.4 | - | 4.2 | ) | 0.86 | ( | 0.74 | - | 0.99 | ) | 266 | 4.2 | ( | 3.7 | - | 4.7 | ) | 0.96 | ( | 0.82 | - | 1.12 | ) |
| **1999** | 364 | 4.1 | ( | 3.7 | - | 4.5 | ) | 1.00 | 362 | 3.6 | ( | 3.3 | - | 4.0 | ) | 0.88 | ( | 0.76 | - | 1.01 | ) | 251 | 4.0 | ( | 3.5 | - | 4.5 | ) | 0.96 | ( | 0.82 | - | 1.13 | ) |
| **2000** | 370 | 4.2 | ( | 3.8 | - | 4.7 | ) | 1.00 | 387 | 3.8 | ( | 3.4 | - | 4.2 | ) | 0.91 | ( | 0.81 | - | 1.03 | ) | 250 | 3.9 | ( | 3.4 | - | 4.4 | ) | 0.94 | ( | 0.82 | - | 1.08 | ) |
| **2001** | 337 | 3.7 | ( | 3.4 | - | 4.2 | ) | 1.00 | 377 | 3.7 | ( | 3.3 | - | 4.1 | ) | 0.97 | ( | 0.84 | - | 1.13 | ) | 231 | 3.6 | ( | 3.2 | - | 4.2 | ) | 0.96 | ( | 0.81 | - | 1.13 | ) |
| **2002** | 304 | 3.4 | ( | 3.0 | - | 3.8 | ) | 1.00 | 323 | 3.1 | ( | 2.8 | - | 3.5 | ) | 0.92 | ( | 0.78 | - | 1.07 | ) | 240 | 3.7 | ( | 3.3 | - | 4.3 | ) | 1.10 | ( | 0.93 | - | 1.30 | ) |
| **2003** | 356 | 4.0 | ( | 3.6 | - | 4.4 | ) | 1.00 | 348 | 3.3 | ( | 3.0 | - | 3.7 | ) | 0.84 | ( | 0.72 | - | 0.97 | ) | 258 | 4.0 | ( | 3.5 | - | 4.5 | ) | 1.01 | ( | 0.86 | - | 1.18 | ) |
| **2004** | 314 | 3.5 | ( | 3.1 | - | 3.9 | ) | 1.00 | 360 | 3.4 | ( | 3.0 | - | 3.7 | ) | 0.97 | ( | 0.84 | - | 1.13 | ) | 238 | 3.7 | ( | 3.2 | - | 4.2 | ) | 1.05 | ( | 0.89 | - | 1.24 | ) |
| **2005** | 338 | 3.8 | ( | 3.4 | - | 4.2 | ) | 1.00 | 349 | 3.2 | ( | 2.9 | - | 3.6 | ) | 0.87 | ( | 0.75 | - | 1.01 | ) | 214 | 3.3 | ( | 2.9 | - | 3.8 | ) | 0.88 | ( | 0.74 | - | 1.04 | ) |
| **2006** | 284 | 3.2 | ( | 2.8 | - | 3.6 | ) | 1.00 | 305 | 2.8 | ( | 2.5 | - | 3.1 | ) | 0.90 | ( | 0.76 | - | 1.05 | ) | 239 | 3.7 | ( | 3.2 | - | 4.2 | ) | 1.17 | ( | 0.98 | - | 1.38 | ) |
| **2007** | 285 | 3.1 | ( | 2.8 | - | 3.5 | ) | 1.00 | 361 | 3.3 | ( | 2.9 | - | 3.6 | ) | 1.05 | ( | 0.90 | - | 1.23 | ) | 180 | 2.7 | ( | 2.3 | - | 3.2 | ) | 0.87 | ( | 0.72 | - | 1.05 | ) |
| **2008** | 324 | 3.6 | ( | 3.2 | - | 4.0 | ) | 1.00 | 351 | 3.2 | ( | 2.8 | - | 3.5 | ) | 0.89 | ( | 0.77 | - | 1.04 | ) | 199 | 2.9 | ( | 2.5 | - | 3.4 | ) | 0.85 | ( | 0.71 | - | 1.01 | ) |
| **2009** | 295 | 3.2 | ( | 2.8 | - | 3.6 | ) | 1.00 | 361 | 3.2 | ( | 2.9 | - | 3.5 | ) | 1.00 | ( | 0.86 | - | 1.16 | ) | 203 | 3.0 | ( | 2.6 | - | 3.5 | ) | 0.95 | ( | 0.79 | - | 1.13 | ) |
| **2010** | 295 | 3.1 | ( | 2.8 | - | 3.5 | ) | 1.00 | 317 | 2.8 | ( | 2.5 | - | 3.1 | ) | 0.87 | ( | 0.75 | - | 1.03 | ) | 224 | 3.4 | ( | 2.9 | - | 3.8 | ) | 1.04 | ( | 0.88 | - | 1.24 | ) |
| **2011** | 286 | 3.1 | ( | 2.8 | - | 3.5 | ) | 1.00 | 358 | 3.1 | ( | 2.8 | - | 3.4 | ) | 1.02 | ( | 0.90 | - | 1.15 | ) | 203 | 3.0 | ( | 2.6 | - | 3.4 | ) | 0.98 | ( | 0.85 | - | 1.13 | ) |
| **2012** | 312 | 3.3 | ( | 3.0 | - | 3.7 | ) | 1.00 | 374 | 3.3 | ( | 2.9 | - | 3.6 | ) | 0.97 | ( | 0.85 | - | 1.11 | ) | 225 | 3.3 | ( | 2.9 | - | 3.8 | ) | 1.00 | ( | 0.86 | - | 1.15 | ) |
| **2013** | 350 | 3.7 | ( | 3.3 | - | 4.1 | ) | 1.00 | 376 | 3.2 | ( | 2.9 | - | 3.6 | ) | 0.87 | ( | 0.76 | - | 1.01 | ) | 221 | 3.2 | ( | 2.8 | - | 3.7 | ) | 0.88 | ( | 0.75 | - | 1.04 | ) |
| **2014** | 293 | 3.0 | ( | 2.7 | - | 3.4 | ) | 1.00 | 392 | 3.3 | ( | 3.0 | - | 3.7 | ) | 1.09 | ( | 0.94 | - | 1.27 | ) | 226 | 3.3 | ( | 2.9 | - | 3.8 | ) | 1.09 | ( | 0.91 | - | 1.29 | ) |
| **2015** | 319 | 3.3 | ( | 2.9 | - | 3.7 | ) | 1.00 | 345 | 2.9 | ( | 2.6 | - | 3.2 | ) | 0.88 | ( | 0.76 | - | 1.03 | ) | 193 | 2.9 | ( | 2.5 | - | 3.3 | ) | 0.86 | ( | 0.72 | - | 1.02 | ) |
| **2016** | 281 | 2.8 | ( | 2.5 | - | 3.2 | ) | 1.00 | 332 | 2.8 | ( | 2.5 | - | 3.1 | ) | 0.96 | ( | 0.82 | - | 1.13 | ) | 202 | 2.9 | ( | 2.5 | - | 3.4 | ) | 1.02 | ( | 0.85 | - | 1.22 | ) |

**Supplemental Table 6 - Post-hoc analysis of suicide methods by population density. Chi square value of the adjusted standardized residuals and p-value. Males and Females**

|  | | Males | | | Females | | |
| --- | --- | --- | --- | --- | --- | --- | --- |
|  |  | population density | | | population density | | |
|  |  | Densely-populated | Intermediate- density | Thinly-populated | Densely-populated | Intermediate- density | Thinly-populated |
| Hanging | ᵡ^2^ | 351.5 | 16.9 | 195.0 | 121.3 | 12.5 | 67.0 |
|  | p | <0.0001* | <0.0001* | <0.0001* | <0.0001* | 0.0004* | <0.0001* |
| Firearms | ᵡ^2^ | 12.2 | 1.2 | 5.0 | 5.6 | 0.0 | 7.1 |
|  | p | 0.0005* | 0.2703 | 0.0249 | 0.0178 | 0.9814 | 0.0079 |
| Fall/jumping from a high place | ᵡ^2^ | 727.2 | 101.8 | 243.1 | 270.1 | 66.6 | 79.3 |
|  | p | <0.0001* | <0.0001* | <0.0001* | <0.0001* | <0.0001* | <0.0001* |
| Poisoning by carbon monoxide | ᵡ^2^ | 1.0 | 1.3 | 0.1 | 0.2 | 0.6 | 0.2 |
|  | p | 0.3169 | 0.2454 | 0.7881 | 0.6680 | 0.4459 | 0.6920 |
| Poisoning by drugs | ᵡ^2^ | 48.1 | 5.8 | 17.8 | 3.0 | 0.0 | 3.0 |
|  | p | <0.0001* | 0.0164 | <0.0001* | 0.0823 | 0.8727 | 0.0810 |
| Poisoning by other substances | ᵡ^2^ | 0.1 | 3.1 | 4.8 | 3.1 | 1.1 | 0.5 |
|  | p | 0.7589 | 0.0786 | 0.0282 | 0.0807 | 0.2930 | 0.4621 |
| Drowning | ᵡ^2^ | 17.0 | 4.9 | 2.7 | 74.2 | 40.0 | 5.4 |
|  | p | <0.0001* | 0.0264 | 0.0982 | <0.0001* | <0.0001* | 0.0201 |
| Jumping/lying before moving object | ᵡ^2^ | 5.0 | 20.0 | 49.0 | 0.8 | 8.3 | 5.2 |
|  | p | 0.0258 | <0.0001* | <0.0001* | 0.3571 | 0.0039 | 0.0228 |
| Cutting and piercing | ᵡ^2^ | 0.7 | 1.7 | 5.1 | 0.9 | 2.7 | 0.7 |
|  | p | 0.3891 | 0.1928 | 0.0247 | 0.3396 | 0.0988 | 0.4073 |
| Other | ᵡ^2^ | 0.8 | 0.1 | 0.3 | 1.0 | 2.3 | 0.4 |
|  | p | 0.3693 | 0.7729 | 0.5687 | 0.3290 | 0.1293 | 0.5145 |

*Significant after Bonferroni correction
