# Supplementary material for: Does population density moderate suicide risk? An Italian population study over the last 30 years
Source: Eur Psychiatry. 2020 Jul 1;63(1):e70. doi: 10.1192/j.eurpsy.2020.69 (PMC7443791; doi:10.1192/j.eurpsy.2020.69)
Supplement: Supplementary file 1 [file S0924933820000693sup001.zip › S0924933820000693supp005.docx]

**Supplemental Table 2 –Suicide by population density, age, and sex. Standardized (Std) rates and Rate Ratios (RR) with corresponding 95% Confidence Intervals (95% CI). Italy, years 2010-2016 (annual average)**

| Age | Population density | |  | Suicide number  2010-2016 | Std rates per 100,000 | RR  (95%CI) | Suicide n.  2010-2016 | Std rates per 100,000 | RR  (95%CI) |  |
| --- | --- | --- | --- | --- | --- | --- | --- | --- | --- | --- |
|  |  | |  | Males | Males | Males | Females | Females | Females |  |
| 15-29 years | Densely-populated | |  | 573 | 5.32 | 1.00 | 157 | 1.51 | 1.00 |  |
|  | Intermediate-density | |  | 817 | 5.80 | 1.09 | 190 | 1.40 | 0.92 |  |
|  |  | |  |  |  | (0.89-1.35) |  |  | (0.78-1.08) |  |
|  | Thinly-populated | |  | 557 | 6.84 | 1.29* | 99 | 1.29 | 0.84 |  |
|  |  | |  |  |  | (1.05-1.58) |  |  | (0.69-1.03) |  |
| *Overall 15-29 years* | | |  | *1948* | *5.92* |  | *446* | *1.41* |  |  |
| 30-54 years | Densely-populated | |  | 2435 | 9.70 | 1.00 | 810 | 3.06 | 1.00 |  |
|  | Intermediate-density | |  | 3776 | 11.32 | 1.17* | 1042 | 3.09 | 1.01 |  |
|  |  | |  |  |  | (1.11-1.23) |  |  | (0.92-1.11) |  |
|  | Thinly-populated | |  | 2496 | 13.15 | 1.36* | 603 | 3.23 | 1.06 |  |
|  |  | |  |  |  | (1.29-1.44) |  |  | (0.95-1.17) |  |
| *Overall 30-54 years* | | |  | *8708* | *11.24* |  | *2455* | *3.11* |  |  |
| 55-69 years | Densely-populated | |  | 1436 | 12.16 | 1.00 | 516 | 3.81 | 1.00 |  |
|  | Intermediate-density | |  | 2175 | 13.99 | 1.15* | 638 | 3.84 | 1.01 |  |
|  |  | |  |  |  | (1.03-1.29) |  |  | (0.92-1.09) |  |
|  | Thinly-populated | |  | 1570 | 16.65 | 1.37* | 382 | 4.00 | 1.05 |  |
|  |  | |  |  |  | (1.22-1.54) |  |  | (0.95-1.15) |  |
| *Overall 55-69 years* | | |  | *5181* | *14.08* |  | *1538* | *3.88* |  |  |
| 70 years and over | Densely-populated | |  | 1684 | 19.44 | 1.00 | 653 | 4.87 | 1.00 |  |
|  | Intermediate-density | |  | 2360 | 22.16 | 1.14* | 624 | 4.05 | 0.83* |  |
|  |  | |  |  |  | (1.09-1.20) |  |  | (0.75-0.93) |  |
|  | Thinly-populated | |  | 1918 | 26.68 | 1.39* | 410 | 4.19 | 0.85* |  |
|  |  | |  |  |  | (1.32-1.46) |  |  | (0.76-0.97) |  |
| *Overall 70 years and over* | | |  | *5963* | *22.36* |  | *1687* | *4.36* |  |  |
|  | |  |  |  |  |  |  |  |  |  |
