# Supplementary material for: Does population density moderate suicide risk? An Italian population study over the last 30 years
Source: Eur Psychiatry. 2020 Jul 1;63(1):e70. doi: 10.1192/j.eurpsy.2020.69 (PMC7443791; doi:10.1192/j.eurpsy.2020.69)
Supplement: Supplementary file 1 [file S0924933820000693sup001.zip › S0924933820000693supp006.docx]

**Supplemental Table 3 –Suicide by population density and geographical macro area. Standardized (Std) rates and Rate Ratios (RR) with corresponding 95% Confidence Intervals (95% CI). Italy, years 2010-2016 (annual average). Males and Females**

|  | Population density | N of municipalities | Suicide n.  2010-2016 | Std rates per 100,000 | RR  (95%CI) | Suicide n.  2010-2016 | Std rates per 100,000 | RR  (95%CI) |  |
| --- | --- | --- | --- | --- | --- | --- | --- | --- | --- |
|  |  |  | Males | Males | Males | Females | Females | Females |  |
| North - West | Densely-populated | 133 | 1,933 | 11.40 | 1.00 | 722 | 3.59 | 1.00 |  |
|  | Intermediate-density | 1029 | 2,781 | 12.95 | 1.15* | 833 | 3.50 | 0.96 |  |
|  |  |  |  |  | (1.08-1.22) |  |  | (0.87-1.06) |  |
|  | Thinly-populated | 1872 | 1,430 | 17.28 | 1.53* | 340 | 3.88 | 1.07 |  |
|  |  |  |  |  | (1.43-1.64) |  |  | (0.94-1.21) |  |
| *Overall North West* | | *3034* | *144* | *13.16* |  | *1895* | *3.60* |  |  |
| North - East | Densely-populated | 19 | 1,283 | 14.36 | 1.00 | 470 | 4.42 | 1.00 |  |
|  | Intermediate-density | 455 | 2,321 | 15.10 | 1.06 | 673 | 3.91 | 0.89 |  |
|  |  |  |  |  | (0.99-1.13) |  |  | (0.79-1.00) |  |
|  | Thinly-populated | 943 | 1,542 | 16.21 | 1.14* | 380 | 3.78 | 0.84* |  |
|  |  |  |  |  | (1.06-1.23) |  |  | (0.74-0.97) |  |
| *Overall North-East* | | *1417* | *5146* | *15.21* |  | *1523* | *4.02* |  |  |
| Centre | Densely-populated | 13 | 1,388 | 11.27 | 1.00 | 477 | 3.18 | 1.00 |  |
|  | Intermediate-density | 224 | 1,537 | 11.92 | 1.06 | 408 | 2.77 | 0.86* |  |
|  |  |  |  |  | (0.98-1.14) |  |  | (0.76-0.99) |  |
|  | Thinly-populated | 738 | 1,300 | 14.24 | 1.27* | 301 | 3.03 | 0.95 |  |
|  |  |  |  |  | (1.18-1.37) |  |  | (0.82-1.10) |  |
| *Overall Centre* | | *975* | *4225* | *12.29* |  | *1186* | *2.99* |  |  |
| South and Islands | Densely-populated | 105 | 1,524 | 8.50 | 1.00 | 467 | 2.25 | 1.00 |  |
|  | Intermediate-density | 593 | 2,489 | 10.74 | 1.26* | 580 | 2.26 | 1.01 |  |
|  |  |  |  |  | (1.18-1.34) |  |  | (0.90-1.12) |  |
|  | Thinly-populated | 1854 | 2,269 | 13.19 | 1.54* | 473 | 2.53 | 1.13* |  |
|  |  |  |  |  | (1.44-1.64) |  |  | (1.01-1.27) |  |
| *Overall South-Islands* | | *2552* | *6282* | *10.82* |  | *1520* | *2.34* |  |  |
| Italy | Densely-populated | 270 | 6,128 | 10.94 | 1.00 | 2,136 | 3.20 | 1.00 |  |
|  | Intermediate-density | 2301 | 9,128 | 12.55 | 1.15* | 2,494 | 3.06 | 0.95 |  |
|  |  |  |  |  | (1.11-1.19) |  |  | (0.90-1.01) |  |
|  | Thinly-populated | 5407 | 6,541 | 14.84 | 1.36* | 1,494 | 3.15 | 0.98 |  |
|  |  |  |  |  | (1.32-1.41) |  |  | (0.91-1.04) |  |
| *Overall Italy* | | *7978* | *21797* | *12.61* |  | *6124* | *3.13* |  |  |
