# Supplementary material for: Does population density moderate suicide risk? An Italian population study over the last 30 years
Source: Eur Psychiatry. 2020 Jul 1;63(1):e70. doi: 10.1192/j.eurpsy.2020.69 (PMC7443791; doi:10.1192/j.eurpsy.2020.69)
Supplement: Supplementary file 1 [file S0924933820000693sup001.zip › S0924933820000693supp007.docx]

**Supplemental Table 4 - Suicide by level of population density: estimated Annual Percentage Change (APC) and Average Annual Percentage Change (AAPC) and corresponding 95% confidence intervals (95% CI), Italy, 1985–2016.**

1. **Males**

| Cohort  Population density | Segment | Lower Endpoint | Upper Endpoint | APC | Lower 95%CI | Upper 95%CI | Test Statistic (t) | Prob > \|t\| | |
| --- | --- | --- | --- | --- | --- | --- | --- | --- | --- |
| Densely populated | 1 | 1985 | 2005 | -2.8* | -3.3 | -2.2 | -10.7 | <0.01 |  |
|  | 2 | 2005 | 2016 | -0.2 | -1.4 | +1.1 | -0.3 | 0.80 |  |
|  | *AAPC* | 1985 | 2016 | -1.8* | -2.4 | -1.3 | -6.7 | <0.01 |  |
| Intermediate-density | 1 | 1985 | 1997 | -0.4 | -1.2 | +0.3 | -1.3 | 0.20 |  |
|  | 2 | 1997 | 2006 | -3.5* | -4.7 | -2.3 | -5.9 | <0.01 |  |
|  | 3 | 2006 | 2012 | +2.2 | -0.3 | +4.8 | +1.8 | 0.10 |  |
|  | 4 | 2012 | 2016 | -3.1 | -6.2 | +0.2 | -2.0 | 0.10 |  |
|  | *AAPC* | 1985 | 2016 | -1.2* | -1.9 | -0.4 | -3.1 | <0.01 |  |
| Thinly-populated | 1 | 1985 | 1998 | -0.8* | -1.3 | -0.2 | -3.1 | <0.01 |  |
|  | 2 | 1998 | 2005 | -2.6* | -4.0 | -1.2 | -3.8 | <0.01 |  |
|  |  | 2005 | 2014 | +0.4 | -0.5 | +1.3 | +0.9 | 0.40 |  |
|  | 3 | 2014 | 2016 | -7.4 | -15.1 | +1.0 | -1.9 | 0.10 |  |
|  | *AAPC* | 1985 | 2016 | -1.3* | -2.0 | -0.6 | -3.7 | <0.01 |  |
| Overall (Italy) | 1 | 1985 | 1997 | -1.0* | -1.6 | -0.3 | -3 | <0.01 |  |
|  | 2 | 1997 | 2006 | -3.1* | -4.2 | -2.0 | -5.8 | <0.01 |  |
|  | 3 | 2006 | 2012 | +1.6 | -0.5 | +3.8 | 1.6 | 0.10 |  |
|  | 4 | 2012 | 2016 | -3.4* | -6.1 | -0.6 | -2.5 | <0.01 |  |
|  | *AAPC* | *1985* | *2016* | *-1.4** | *-2.1* | *-0.8* | *-4.3* | *<0.01* |  |

1. **Females**

| Cohort  Population density | Segment | Lower Endpoint | Upper Endpoint | APC | Lower 95%CI | Upper 95% CI | Test Statistic (t) | Prob > \|t\| |  |
| --- | --- | --- | --- | --- | --- | --- | --- | --- | --- |
| Densely populated | 1 | 1985 | 2007 | -3.8* | -4.2 | -3.4 | -19.7 | <0.01 |  |
|  | 2 | 2007 | 2013 | +1.4 | -3.0 | +6.0 | +0.7 | 0.50 |  |
|  | 3 | 2013 | 2016 | -5.5 | -14.8 | +4.9 | -1.1 | 0.30 |  |
|  | *AAPC* | 1985 | 2016 | -3.0* | -4.2 | -1.7 | -4.6 | <0.01 |  |
| Intermediate-density | 1 | 1985 | 2006 | -2.6* | -3.1 | -2.1 | -10.8 | <0.01 |  |
|  | 2 | 2006 | 2016 | -0.4 | -2.0 | +1.2 | -0.5 | 0.60 |  |
|  | *AAPC* | 1985 | 2016 | -1.9* | -2.5 | -1.3 | -6.4 | <0.01 |  |
| Thinly-populated | 1 | 1985 | 2008 | -2.5* | -2.9 | -2.1 | -12.4 | <0.01 |  |
|  | 2 | 2008 | 2016 | -0.5 | -2.8 | +1.8 | -0.4 | 0.70 |  |
|  | *AAPC* | 1985 | 2016 | -2.0* | -2.6 | -1.4 | -6.1 | <0.01 |  |
| Overall (Italy) | 1 | 1985 | 2007 | -3.0* | -3.3 | -2.7 | -20.3 | <0.01 |  |
|  | 2 | 2007 | 2016 | -0.4 | -1.7 | 0.9 | -0.6 | 0.60 |  |
|  | *AAPC* | *1985* | *2016* | *-2.2** | *-2.6* | *-1.8* | *-10.5* | *<0.01* |  |
