# Supplementary material for: Does population density moderate suicide risk? An Italian population study over the last 30 years
Source: Eur Psychiatry. 2020 Jul 1;63(1):e70. doi: 10.1192/j.eurpsy.2020.69 (PMC7443791; doi:10.1192/j.eurpsy.2020.69)
Supplement: Supplementary file 1 [file S0924933820000693sup001.zip › S0924933820000693supp008.docx]

**Supplemental Table 5 - Suicide by population density of area of residence and sex. Number of suicides (n), standardized (std) rate, and Rate Ratios (RR) with corresponding 95% Confidence Intervals (95% CI). Italy, years 1985-2016**

**MALES**

|  | **Population density** | | | | | | | | | | | | | | | | | | | | | | | | | | | | | | | | | |
| --- | --- | --- | --- | --- | --- | --- | --- | --- | --- | --- | --- | --- | --- | --- | --- | --- | --- | --- | --- | --- | --- | --- | --- | --- | --- | --- | --- | --- | --- | --- | --- | --- | --- | --- |
|  | **Densely populated** | | | | | | | | **Intermediate density** | | | | | | | | | | | |  | **Thinly populated** | | | | | | | | | | | |  |
| **Year** | **n** | **Std rate** | **95%CI** | | | | | **RR** | **n** | **Std rate** | **95%CI** | | | | | **RR** | **95%CI** | | | | | **n** | **Std rate** | **95%CI** | | | | | **RR** | **95%CI** | | | | |
| **1985** | 1130 | 18.4 | ( | 17.2 | - | 19.9 | ) | 1.00 | 1239 | 19.2 | ( | 17.7 | - | 21.1 | ) | 1.04 | ( | 0.96 | - | 1.12 | ) | 950 | 20.5 | ( | 18.1 | - | 25.2 | ) | 1.13 | ( | 1.03 | - | 1.23 | ) |
| **1986** | 1108 | 18.0 | ( | 16.7 | - | 19.5 | ) | 1.00 | 1154 | 17.1 | ( | 15.9 | - | 18.8 | ) | 0.96 | ( | 0.87 | - | 1.06 | ) | 980 | 20.8 | ( | 18.6 | - | 24.6 | ) | 1.18 | ( | 1.06 | - | 1.31 | ) |
| **1987** | 1099 | 17.9 | ( | 16.6 | - | 19.5 | ) | 1.00 | 1134 | 16.4 | ( | 15.3 | - | 18.0 | ) | 0.96 | ( | 0.88 | - | 1.04 | ) | 984 | 20.1 | ( | 18.8 | - | 22.9 | ) | 1.21 | ( | 1.11 | - | 1.31 | ) |
| **1988** | 1004 | 16.6 | ( | 15.3 | - | 18.1 | ) | 1.00 | 1148 | 17.0 | ( | 15.8 | - | 18.5 | ) | 1.06 | ( | 0.97 | - | 1.15 | ) | 906 | 18.6 | ( | 17.3 | - | 21.0 | ) | 1.21 | ( | 1.11 | - | 1.33 | ) |
| **1989** | 1087 | 16.7 | ( | 15.6 | - | 18.0 | ) | 1.00 | 1114 | 15.3 | ( | 14.3 | - | 16.6 | ) | 0.94 | ( | 0.86 | - | 1.02 | ) | 887 | 18.9 | ( | 17.2 | - | 21.5 | ) | 1.10 | ( | 1.01 | - | 1.21 | ) |
| **1990** | 1069 | 16.5 | ( | 15.4 | - | 17.8 | ) | 1.00 | 1164 | 16.4 | ( | 15.3 | - | 17.7 | ) | 0.99 | ( | 0.91 | - | 1.07 | ) | 908 | 18.2 | ( | 16.9 | - | 20.0 | ) | 1.13 | ( | 1.04 | - | 1.24 | ) |
| **1991** | 1004 | 15.3 | ( | 14.2 | - | 16.5 | ) | 1.00 | 1183 | 15.7 | ( | 14.7 | - | 16.8 | ) | 1.06 | ( | 0.97 | - | 1.15 | ) | 976 | 18.9 | ( | 17.7 | - | 20.4 | ) | 1.29 | ( | 1.18 | - | 1.41 | ) |
| **1992** | 1059 | 15.7 | ( | 14.6 | - | 16.9 | ) | 1.00 | 1248 | 16.0 | ( | 15.1 | - | 17.1 | ) | 1.05 | ( | 0.96 | - | 1.14 | ) | 969 | 18.4 | ( | 17.2 | - | 19.7 | ) | 1.20 | ( | 1.10 | - | 1.31 | ) |
| **1993** | 1136 | 17.2 | ( | 16.0 | - | 18.5 | ) | 1.00 | 1307 | 16.6 | ( | 15.6 | - | 17.7 | ) | 1.01 | ( | 0.94 | - | 1.10 | ) | 999 | 18.8 | ( | 17.6 | - | 20.1 | ) | 1.15 | ( | 1.06 | - | 1.25 | ) |
| **1994** | 1052 | 15.2 | ( | 14.1 | - | 16.3 | ) | 1.00 | 1298 | 16.4 | ( | 15.5 | - | 17.5 | ) | 1.08 | ( | 0.99 | - | 1.17 | ) | 993 | 18.3 | ( | 17.1 | - | 19.6 | ) | 1.23 | ( | 1.13 | - | 1.34 | ) |
| **1995** | 1064 | 15.0 | ( | 14.0 | - | 16.1 | ) | 1.00 | 1320 | 16.5 | ( | 15.5 | - | 17.6 | ) | 1.07 | ( | 0.99 | - | 1.17 | ) | 987 | 18.3 | ( | 17.1 | - | 19.6 | ) | 1.21 | ( | 1.11 | - | 1.31 | ) |
| **1996** | 1046 | 15.1 | ( | 14.1 | - | 16.2 | ) | 1.00 | 1373 | 16.7 | ( | 15.7 | - | 17.8 | ) | 1.13 | ( | 1.04 | - | 1.22 | ) | 986 | 17.7 | ( | 16.6 | - | 19.0 | ) | 1.23 | ( | 1.13 | - | 1.34 | ) |
| **1997** | 1080 | 15.5 | ( | 14.5 | - | 16.6 | ) | 1.00 | 1398 | 16.7 | ( | 15.8 | - | 17.8 | ) | 1.10 | ( | 1.02 | - | 1.20 | ) | 1031 | 18.8 | ( | 17.6 | - | 20.1 | ) | 1.24 | ( | 1.14 | - | 1.35 | ) |
| **1998** | 1033 | 14.7 | ( | 13.8 | - | 15.8 | ) | 1.00 | 1269 | 15.2 | ( | 14.3 | - | 16.2 | ) | 1.04 | ( | 0.94 | - | 1.14 | ) | 1055 | 19.0 | ( | 17.8 | - | 20.2 | ) | 1.30 | ( | 1.17 | - | 1.44 | ) |
| **1999** | 853 | 12.0 | ( | 11.1 | - | 13.0 | ) | 1.00 | 1232 | 14.5 | ( | 13.7 | - | 15.5 | ) | 1.21 | ( | 1.11 | - | 1.32 | ) | 917 | 16.4 | ( | 15.3 | - | 17.5 | ) | 1.38 | ( | 1.25 | - | 1.51 | ) |
| **2000** | 846 | 11.9 | ( | 11.1 | - | 12.9 | ) | 1.00 | 1196 | 13.8 | ( | 13.0 | - | 14.7 | ) | 1.17 | ( | 1.07 | - | 1.28 | ) | 900 | 16.1 | ( | 15.0 | - | 17.2 | ) | 1.36 | ( | 1.24 | - | 1.49 | ) |
| **2001** | 773 | 10.7 | ( | 9.9 | - | 11.6 | ) | 1.00 | 1225 | 14.1 | ( | 13.3 | - | 15.0 | ) | 1.30 | ( | 1.19 | - | 1.43 | ) | 906 | 16.1 | ( | 15.0 | - | 17.2 | ) | 1.50 | ( | 1.36 | - | 1.65 | ) |
| **2002** | 830 | 11.5 | ( | 10.7 | - | 12.4 | ) | 1.00 | 1240 | 14.0 | ( | 13.2 | - | 14.9 | ) | 1.22 | ( | 1.12 | - | 1.33 | ) | 942 | 16.5 | ( | 15.5 | - | 17.7 | ) | 1.45 | ( | 1.32 | - | 1.59 | ) |
| **2003** | 847 | 11.5 | ( | 10.7 | - | 12.4 | ) | 1.00 | 1155 | 13.3 | ( | 12.5 | - | 14.1 | ) | 1.11 | ( | 1.01 | - | 1.21 | ) | 948 | 16.6 | ( | 15.5 | - | 17.7 | ) | 1.42 | ( | 1.29 | - | 1.56 | ) |
| **2004** | 831 | 11.5 | ( | 10.7 | - | 12.4 | ) | 1.00 | 1168 | 12.9 | ( | 12.1 | - | 13.7 | ) | 1.13 | ( | 1.03 | - | 1.24 | ) | 915 | 15.8 | ( | 14.8 | - | 16.9 | ) | 1.39 | ( | 1.27 | - | 1.53 | ) |
| **2005** | 766 | 10.4 | ( | 9.6 | - | 11.2 | ) | 1.00 | 1078 | 11.6 | ( | 10.9 | - | 12.4 | ) | 1.12 | ( | 1.04 | - | 1.21 | ) | 838 | 14.2 | ( | 13.2 | - | 15.2 | ) | 1.38 | ( | 1.27 | - | 1.50 | ) |
| **2006** | 774 | 10.4 | ( | 9.7 | - | 11.2 | ) | 1.00 | 1076 | 11.5 | ( | 10.8 | - | 12.3 | ) | 1.10 | ( | 1.00 | - | 1.21 | ) | 881 | 15.0 | ( | 14.0 | - | 16.1 | ) | 1.43 | ( | 1.30 | - | 1.58 | ) |
| **2007** | 777 | 10.4 | ( | 9.6 | - | 11.2 | ) | 1.00 | 1136 | 12.0 | ( | 11.3 | - | 12.7 | ) | 1.15 | ( | 1.05 | - | 1.26 | ) | 846 | 14.2 | ( | 13.3 | - | 15.3 | ) | 1.36 | ( | 1.24 | - | 1.50 | ) |
| **2008** | 788 | 10.3 | ( | 9.6 | - | 11.1 | ) | 1.00 | 1158 | 12.0 | ( | 11.3 | - | 12.7 | ) | 1.14 | ( | 1.04 | - | 1.25 | ) | 919 | 15.3 | ( | 14.3 | - | 16.4 | ) | 1.46 | ( | 1.33 | - | 1.60 | ) |
| **2009** | 849 | 11.1 | ( | 10.4 | - | 12.0 | ) | 1.00 | 1247 | 12.7 | ( | 11.9 | - | 13.4 | ) | 1.14 | ( | 1.04 | - | 1.24 | ) | 914 | 14.9 | ( | 14.0 | - | 16.0 | ) | 1.35 | ( | 1.23 | - | 1.48 | ) |
| **2010** | 885 | 11.6 | ( | 10.8 | - | 12.4 | ) | 1.00 | 1229 | 12.3 | ( | 11.6 | - | 13.1 | ) | 1.07 | ( | 0.99 | - | 1.15 | ) | 913 | 14.9 | ( | 13.9 | - | 15.9 | ) | 1.29 | ( | 1.20 | - | 1.40 | ) |
| **2011** | 851 | 11.0 | ( | 10.3 | - | 11.8 | ) | 1.00 | 1334 | 13.2 | ( | 12.5 | - | 14.0 | ) | 1.20 | ( | 1.10 | - | 1.31 | ) | 997 | 16.1 | ( | 15.1 | - | 17.1 | ) | 1.47 | ( | 1.34 | - | 1.61 | ) |
| **2012** | 917 | 11.7 | ( | 10.9 | - | 12.5 | ) | 1.00 | 1355 | 13.3 | ( | 12.5 | - | 14.0 | ) | 1.13 | ( | 1.04 | - | 1.23 | ) | 955 | 15.3 | ( | 14.3 | - | 16.3 | ) | 1.31 | ( | 1.20 | - | 1.43 | ) |
| **2013** | 909 | 11.4 | ( | 10.6 | - | 12.2 | ) | 1.00 | 1376 | 13.1 | ( | 12.4 | - | 13.9 | ) | 1.17 | ( | 1.07 | - | 1.27 | ) | 940 | 14.9 | ( | 14.0 | - | 15.9 | ) | 1.32 | ( | 1.21 | - | 1.45 | ) |
| **2014** | 886 | 10.8 | ( | 10.1 | - | 11.6 | ) | 1.00 | 1264 | 12.0 | ( | 11.4 | - | 12.8 | ) | 1.11 | ( | 1.03 | - | 1.19 | ) | 982 | 15.5 | ( | 14.5 | - | 16.5 | ) | 1.43 | ( | 1.33 | - | 1.55 | ) |
| **2015** | 839 | 10.1 | ( | 9.4 | - | 10.8 | ) | 1.00 | 1297 | 12.2 | ( | 11.5 | - | 12.9 | ) | 1.20 | ( | 1.11 | - | 1.30 | ) | 903 | 14.1 | ( | 13.2 | - | 15.1 | ) | 1.40 | ( | 1.28 | - | 1.52 | ) |
| **2016** | 841 | 10.1 | ( | 9.4 | - | 10.8 | ) | 1.00 | 1273 | 11.8 | ( | 11.1 | - | 12.5 | ) | 1.17 | ( | 1.08 | - | 1.28 | ) | 851 | 13.2 | ( | 12.4 | - | 14.2 | ) | 1.32 | ( | 1.20 | - | 1.45 | ) |

**FEMALES**

| **Population density** | | | | | | | | | | | | | | | | | | | | | | | | | | | | | | | | | | |
| --- | --- | --- | --- | --- | --- | --- | --- | --- | --- | --- | --- | --- | --- | --- | --- | --- | --- | --- | --- | --- | --- | --- | --- | --- | --- | --- | --- | --- | --- | --- | --- | --- | --- | --- |
|  | **Densely populated** | | | | | | | | **Intermediate density** | | | | | | | | | | | |  | **Thinly populated** | | | | | | | | | | | |  |
| **Year** | **n** | **Std rate** | **95%CI** | | | | | **RR** | **n** | **Std rate** | **95%CI** | | | | | **RR** | **95%CI** | | | | | **n** | **Std rate** | **95%CI** | | | | | **RR** | **95%CI** | | | | |
| **1985** | 614 | 7.6 | ( | 7.0 | - | 8.3 | ) | 1.00 | 437 | 5.4 | ( | 4.9 | - | 6.0 | ) | 0.71 | ( | 0.63 | - | 0.81 | ) | 324 | 5.8 | ( | 5.2 | - | 6.6 | ) | 0.78 | ( | 0.68 | - | 0.89 | ) |
| **1986** | 599 | 7.3 | ( | 6.7 | - | 7.9 | ) | 1.00 | 450 | 5.5 | ( | 5.0 | - | 6.1 | ) | 0.75 | ( | 0.66 | - | 0.84 | ) | 333 | 6.0 | ( | 5.3 | - | 6.8 | ) | 0.82 | ( | 0.72 | - | 0.94 | ) |
| **1987** | 531 | 6.5 | ( | 5.9 | - | 7.1 | ) | 1.00 | 436 | 5.2 | ( | 4.7 | - | 5.8 | ) | 0.81 | ( | 0.71 | - | 0.92 | ) | 275 | 4.9 | ( | 4.3 | - | 5.6 | ) | 0.76 | ( | 0.66 | - | 0.88 | ) |
| **1988** | 551 | 6.6 | ( | 6.0 | - | 7.2 | ) | 1.00 | 422 | 4.9 | ( | 4.5 | - | 5.5 | ) | 0.75 | ( | 0.66 | - | 0.85 | ) | 302 | 5.3 | ( | 4.7 | - | 6.1 | ) | 0.81 | ( | 0.70 | - | 0.93 | ) |
| **1989** | 564 | 6.7 | ( | 6.1 | - | 7.3 | ) | 1.00 | 369 | 4.2 | ( | 3.8 | - | 4.7 | ) | 0.63 | ( | 0.55 | - | 0.72 | ) | 274 | 4.8 | ( | 4.3 | - | 5.5 | ) | 0.72 | ( | 0.62 | - | 0.83 | ) |
| **1990** | 500 | 5.8 | ( | 5.3 | - | 6.4 | ) | 1.00 | 419 | 4.7 | ( | 4.2 | - | 5.2 | ) | 0.80 | ( | 0.70 | - | 0.91 | ) | 282 | 4.9 | ( | 4.4 | - | 5.6 | ) | 0.83 | ( | 0.72 | - | 0.96 | ) |
| **1991** | 482 | 5.6 | ( | 5.1 | - | 6.1 | ) | 1.00 | 459 | 5.1 | ( | 4.7 | - | 5.7 | ) | 0.90 | ( | 0.81 | - | 1.00 | ) | 297 | 5.0 | ( | 4.4 | - | 5.6 | ) | 0.89 | ( | 0.79 | - | 1.01 | ) |
| **1992** | 491 | 5.7 | ( | 5.2 | - | 6.3 | ) | 1.00 | 414 | 4.5 | ( | 4.1 | - | 5.0 | ) | 0.79 | ( | 0.71 | - | 0.89 | ) | 287 | 4.8 | ( | 4.2 | - | 5.4 | ) | 0.84 | ( | 0.74 | - | 0.95 | ) |
| **1993** | 468 | 5.4 | ( | 4.9 | - | 6.0 | ) | 1.00 | 397 | 4.3 | ( | 3.9 | - | 4.8 | ) | 0.79 | ( | 0.72 | - | 0.88 | ) | 289 | 4.7 | ( | 4.2 | - | 5.4 | ) | 0.88 | ( | 0.79 | - | 0.98 | ) |
| **1994** | 426 | 4.9 | ( | 4.4 | - | 5.4 | ) | 1.00 | 413 | 4.4 | ( | 4.0 | - | 4.9 | ) | 0.89 | ( | 0.78 | - | 1.02 | ) | 254 | 4.2 | ( | 3.7 | - | 4.7 | ) | 0.85 | ( | 0.73 | - | 0.99 | ) |
| **1995** | 430 | 4.9 | ( | 4.5 | - | 5.4 | ) | 1.00 | 412 | 4.3 | ( | 3.9 | - | 4.8 | ) | 0.88 | ( | 0.77 | - | 1.01 | ) | 283 | 4.6 | ( | 4.0 | - | 5.2 | ) | 0.94 | ( | 0.81 | - | 1.09 | ) |
| **1996** | 479 | 5.5 | ( | 5.0 | - | 6.0 | ) | 1.00 | 457 | 4.8 | ( | 4.4 | - | 5.3 | ) | 0.87 | ( | 0.76 | - | 0.99 | ) | 277 | 4.5 | ( | 4.0 | - | 5.1 | ) | 0.82 | ( | 0.71 | - | 0.95 | ) |
| **1997** | 404 | 4.6 | ( | 4.1 | - | 5.0 | ) | 1.00 | 434 | 4.4 | ( | 4.0 | - | 4.9 | ) | 0.96 | ( | 0.84 | - | 1.10 | ) | 296 | 4.7 | ( | 4.2 | - | 5.3 | ) | 1.03 | ( | 0.89 | - | 1.20 | ) |
| **1998** | 388 | 4.4 | ( | 4.0 | - | 4.9 | ) | 1.00 | 374 | 3.8 | ( | 3.4 | - | 4.2 | ) | 0.86 | ( | 0.74 | - | 0.99 | ) | 266 | 4.2 | ( | 3.7 | - | 4.7 | ) | 0.96 | ( | 0.82 | - | 1.12 | ) |
| **1999** | 364 | 4.1 | ( | 3.7 | - | 4.5 | ) | 1.00 | 362 | 3.6 | ( | 3.3 | - | 4.0 | ) | 0.88 | ( | 0.76 | - | 1.01 | ) | 251 | 4.0 | ( | 3.5 | - | 4.5 | ) | 0.96 | ( | 0.82 | - | 1.13 | ) |
| **2000** | 370 | 4.2 | ( | 3.8 | - | 4.7 | ) | 1.00 | 387 | 3.8 | ( | 3.4 | - | 4.2 | ) | 0.91 | ( | 0.81 | - | 1.03 | ) | 250 | 3.9 | ( | 3.4 | - | 4.4 | ) | 0.94 | ( | 0.82 | - | 1.08 | ) |
| **2001** | 337 | 3.7 | ( | 3.4 | - | 4.2 | ) | 1.00 | 377 | 3.7 | ( | 3.3 | - | 4.1 | ) | 0.97 | ( | 0.84 | - | 1.13 | ) | 231 | 3.6 | ( | 3.2 | - | 4.2 | ) | 0.96 | ( | 0.81 | - | 1.13 | ) |
| **2002** | 304 | 3.4 | ( | 3.0 | - | 3.8 | ) | 1.00 | 323 | 3.1 | ( | 2.8 | - | 3.5 | ) | 0.92 | ( | 0.78 | - | 1.07 | ) | 240 | 3.7 | ( | 3.3 | - | 4.3 | ) | 1.10 | ( | 0.93 | - | 1.30 | ) |
| **2003** | 356 | 4.0 | ( | 3.6 | - | 4.4 | ) | 1.00 | 348 | 3.3 | ( | 3.0 | - | 3.7 | ) | 0.84 | ( | 0.72 | - | 0.97 | ) | 258 | 4.0 | ( | 3.5 | - | 4.5 | ) | 1.01 | ( | 0.86 | - | 1.18 | ) |
| **2004** | 314 | 3.5 | ( | 3.1 | - | 3.9 | ) | 1.00 | 360 | 3.4 | ( | 3.0 | - | 3.7 | ) | 0.97 | ( | 0.84 | - | 1.13 | ) | 238 | 3.7 | ( | 3.2 | - | 4.2 | ) | 1.05 | ( | 0.89 | - | 1.24 | ) |
| **2005** | 338 | 3.8 | ( | 3.4 | - | 4.2 | ) | 1.00 | 349 | 3.2 | ( | 2.9 | - | 3.6 | ) | 0.87 | ( | 0.75 | - | 1.01 | ) | 214 | 3.3 | ( | 2.9 | - | 3.8 | ) | 0.88 | ( | 0.74 | - | 1.04 | ) |
| **2006** | 284 | 3.2 | ( | 2.8 | - | 3.6 | ) | 1.00 | 305 | 2.8 | ( | 2.5 | - | 3.1 | ) | 0.90 | ( | 0.76 | - | 1.05 | ) | 239 | 3.7 | ( | 3.2 | - | 4.2 | ) | 1.17 | ( | 0.98 | - | 1.38 | ) |
| **2007** | 285 | 3.1 | ( | 2.8 | - | 3.5 | ) | 1.00 | 361 | 3.3 | ( | 2.9 | - | 3.6 | ) | 1.05 | ( | 0.90 | - | 1.23 | ) | 180 | 2.7 | ( | 2.3 | - | 3.2 | ) | 0.87 | ( | 0.72 | - | 1.05 | ) |
| **2008** | 324 | 3.6 | ( | 3.2 | - | 4.0 | ) | 1.00 | 351 | 3.2 | ( | 2.8 | - | 3.5 | ) | 0.89 | ( | 0.77 | - | 1.04 | ) | 199 | 2.9 | ( | 2.5 | - | 3.4 | ) | 0.85 | ( | 0.71 | - | 1.01 | ) |
| **2009** | 295 | 3.2 | ( | 2.8 | - | 3.6 | ) | 1.00 | 361 | 3.2 | ( | 2.9 | - | 3.5 | ) | 1.00 | ( | 0.86 | - | 1.16 | ) | 203 | 3.0 | ( | 2.6 | - | 3.5 | ) | 0.95 | ( | 0.79 | - | 1.13 | ) |
| **2010** | 295 | 3.1 | ( | 2.8 | - | 3.5 | ) | 1.00 | 317 | 2.8 | ( | 2.5 | - | 3.1 | ) | 0.87 | ( | 0.75 | - | 1.03 | ) | 224 | 3.4 | ( | 2.9 | - | 3.8 | ) | 1.04 | ( | 0.88 | - | 1.24 | ) |
| **2011** | 286 | 3.1 | ( | 2.8 | - | 3.5 | ) | 1.00 | 358 | 3.1 | ( | 2.8 | - | 3.4 | ) | 1.02 | ( | 0.90 | - | 1.15 | ) | 203 | 3.0 | ( | 2.6 | - | 3.4 | ) | 0.98 | ( | 0.85 | - | 1.13 | ) |
| **2012** | 312 | 3.3 | ( | 3.0 | - | 3.7 | ) | 1.00 | 374 | 3.3 | ( | 2.9 | - | 3.6 | ) | 0.97 | ( | 0.85 | - | 1.11 | ) | 225 | 3.3 | ( | 2.9 | - | 3.8 | ) | 1.00 | ( | 0.86 | - | 1.15 | ) |
| **2013** | 350 | 3.7 | ( | 3.3 | - | 4.1 | ) | 1.00 | 376 | 3.2 | ( | 2.9 | - | 3.6 | ) | 0.87 | ( | 0.76 | - | 1.01 | ) | 221 | 3.2 | ( | 2.8 | - | 3.7 | ) | 0.88 | ( | 0.75 | - | 1.04 | ) |
| **2014** | 293 | 3.0 | ( | 2.7 | - | 3.4 | ) | 1.00 | 392 | 3.3 | ( | 3.0 | - | 3.7 | ) | 1.09 | ( | 0.94 | - | 1.27 | ) | 226 | 3.3 | ( | 2.9 | - | 3.8 | ) | 1.09 | ( | 0.91 | - | 1.29 | ) |
| **2015** | 319 | 3.3 | ( | 2.9 | - | 3.7 | ) | 1.00 | 345 | 2.9 | ( | 2.6 | - | 3.2 | ) | 0.88 | ( | 0.76 | - | 1.03 | ) | 193 | 2.9 | ( | 2.5 | - | 3.3 | ) | 0.86 | ( | 0.72 | - | 1.02 | ) |
| **2016** | 281 | 2.8 | ( | 2.5 | - | 3.2 | ) | 1.00 | 332 | 2.8 | ( | 2.5 | - | 3.1 | ) | 0.96 | ( | 0.82 | - | 1.13 | ) | 202 | 2.9 | ( | 2.5 | - | 3.4 | ) | 1.02 | ( | 0.85 | - | 1.22 | ) |
