# Supplementary material for: Does population density moderate suicide risk? An Italian population study over the last 30 years
Source: Eur Psychiatry. 2020 Jul 1;63(1):e70. doi: 10.1192/j.eurpsy.2020.69 (PMC7443791; doi:10.1192/j.eurpsy.2020.69)
Supplement: Supplementary file 1 [file S0924933820000693sup001.zip › S0924933820000693supp009.docx]

**Supplemental Table 6 - Post-hoc analysis of suicide methods by population density. Chi square value of the adjusted standardized residuals and p-value. Males and Females**

|  | | Males | | | Females | | |
| --- | --- | --- | --- | --- | --- | --- | --- |
|  |  | population density | | | population density | | |
|  |  | Densely-populated | Intermediate- density | Thinly-populated | Densely-populated | Intermediate- density | Thinly-populated |
| Hanging | ᵡ^2^ | 351.5 | 16.9 | 195.0 | 121.3 | 12.5 | 67.0 |
|  | p | <0.0001* | <0.0001* | <0.0001* | <0.0001* | 0.0004* | <0.0001* |
| Firearms | ᵡ^2^ | 12.2 | 1.2 | 5.0 | 5.6 | 0.0 | 7.1 |
|  | p | 0.0005* | 0.2703 | 0.0249 | 0.0178 | 0.9814 | 0.0079 |
| Fall/jumping from a high place | ᵡ^2^ | 727.2 | 101.8 | 243.1 | 270.1 | 66.6 | 79.3 |
|  | p | <0.0001* | <0.0001* | <0.0001* | <0.0001* | <0.0001* | <0.0001* |
| Poisoning by carbon monoxide | ᵡ^2^ | 1.0 | 1.3 | 0.1 | 0.2 | 0.6 | 0.2 |
|  | p | 0.3169 | 0.2454 | 0.7881 | 0.6680 | 0.4459 | 0.6920 |
| Poisoning by drugs | ᵡ^2^ | 48.1 | 5.8 | 17.8 | 3.0 | 0.0 | 3.0 |
|  | p | <0.0001* | 0.0164 | <0.0001* | 0.0823 | 0.8727 | 0.0810 |
| Poisoning by other substances | ᵡ^2^ | 0.1 | 3.1 | 4.8 | 3.1 | 1.1 | 0.5 |
|  | p | 0.7589 | 0.0786 | 0.0282 | 0.0807 | 0.2930 | 0.4621 |
| Drowning | ᵡ^2^ | 17.0 | 4.9 | 2.7 | 74.2 | 40.0 | 5.4 |
|  | p | <0.0001* | 0.0264 | 0.0982 | <0.0001* | <0.0001* | 0.0201 |
| Jumping/lying before moving object | ᵡ^2^ | 5.0 | 20.0 | 49.0 | 0.8 | 8.3 | 5.2 |
|  | p | 0.0258 | <0.0001* | <0.0001* | 0.3571 | 0.0039 | 0.0228 |
| Cutting and piercing | ᵡ^2^ | 0.7 | 1.7 | 5.1 | 0.9 | 2.7 | 0.7 |
|  | p | 0.3891 | 0.1928 | 0.0247 | 0.3396 | 0.0988 | 0.4073 |
| Other | ᵡ^2^ | 0.8 | 0.1 | 0.3 | 1.0 | 2.3 | 0.4 |
|  | p | 0.3693 | 0.7729 | 0.5687 | 0.3290 | 0.1293 | 0.5145 |

*Significant after Bonferroni correction
