# Supplementary figures and images for: Does population density moderate suicide risk? An Italian population study over the last 30 years
Source: Eur Psychiatry. 2020 Jul 1;63(1):e70. doi: 10.1192/j.eurpsy.2020.69 (PMC7443791; doi:10.1192/j.eurpsy.2020.69)

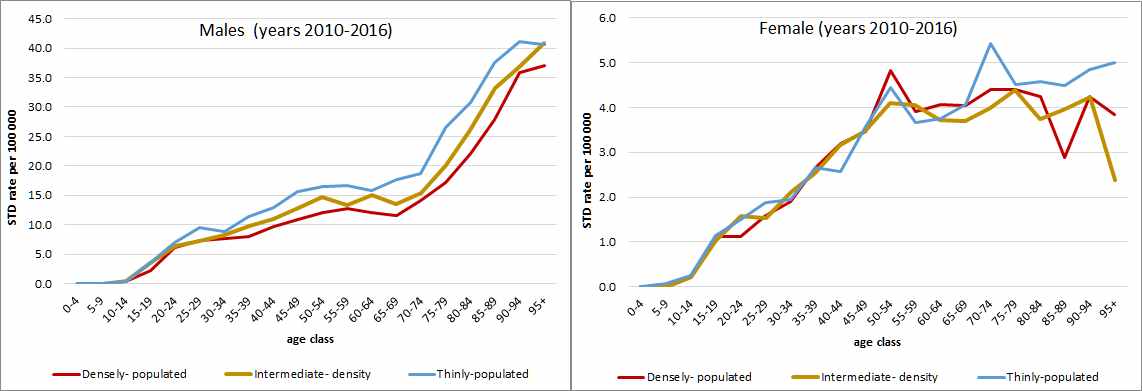

Supplement: Supplementary file 1 [file S0924933820000693sup001.zip › S0924933820000693supp001.jpg]

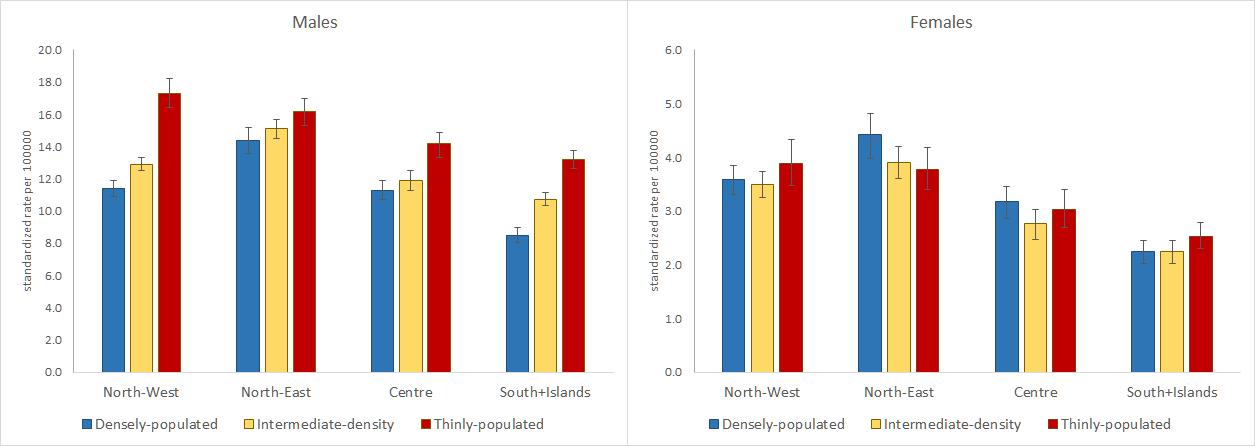

Supplement: Supplementary file 1 [file S0924933820000693sup001.zip › S0924933820000693supp002.jpg]

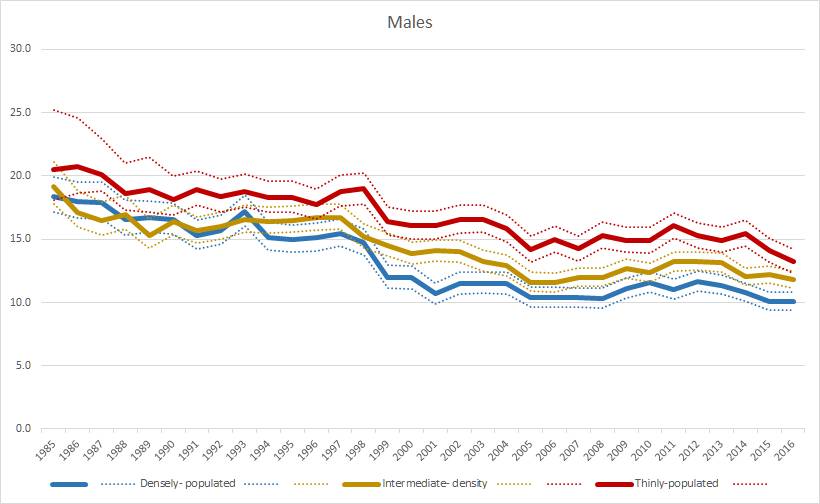

Supplement: Supplementary file 1 [file S0924933820000693sup001.zip › S0924933820000693supp003.jpg]
